# Synaptic zinc potentiates AMPA receptor function in mouse auditory cortex

**DOI:** 10.1016/j.celrep.2023.112932

**Published:** 2023-08-15

**Authors:** Philip T.R. Bender, Mason McCollum, Helen Boyd-Pratt, Benjamin Z. Mendelson, Charles T. Anderson

**Affiliations:** 1 Department of Neuroscience, Rockefeller Neuroscience Institute, West Virginia University School of Medicine, Morgantown, WV 26506, USA; 2 Lead contact

## Abstract

Synaptic zinc signaling modulates synaptic activity and is present in specific populations of cortical neurons, suggesting that synaptic zinc contributes to the diversity of intracortical synaptic microcircuits and their functional specificity. To understand the role of zinc signaling in the cortex, we performed whole-cell patch-clamp recordings from intratelencephalic (IT)-type neurons and pyramidal tract (PT)-type neurons in layer 5 of the mouse auditory cortex during optogenetic stimulation of specific classes of presynaptic neurons. Our results show that synaptic zinc potentiates AMPA receptor (AMPAR) function in a synapse-specific manner. We performed *in vivo* 2-photon calcium imaging of the same classes of neurons in awake mice and found that changes in synaptic zinc can widen or sharpen the sound-frequency tuning bandwidth of IT-type neurons but only widen the tuning bandwidth of PT-type neurons. These results provide evidence for synapse- and cell-type-specific actions of synaptic zinc in the cortex.

## In brief

Bender et al. provide evidence that synaptically released zinc potentiates AMPAR function in a synapse-specific manner, and that this potentiation can be enhanced or suppressed in a zinc concentration-dependent manner. These findings unify opposing reports of the function of synaptic zinc signaling.

## Graphical Abstract

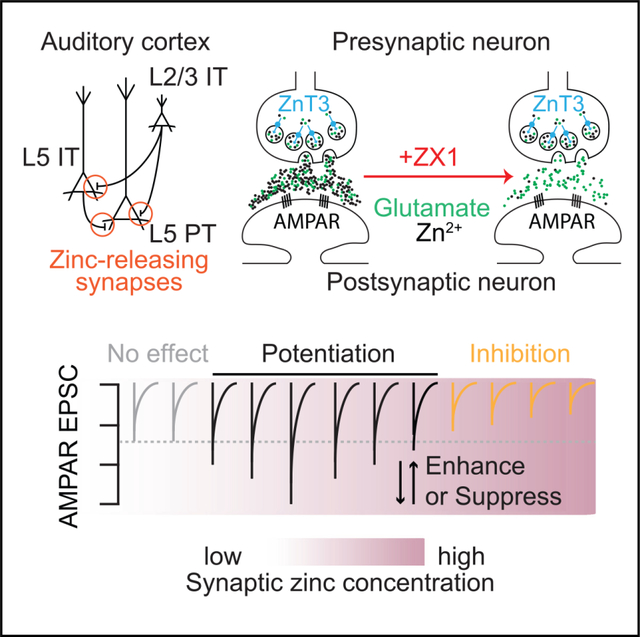

## INTRODUCTION

In the brain, many glutamatergic neurons corelease glutamate and zinc ions from the same synaptic vesicles during synaptic transmission.^1,2^ These zincergic neurons are widespread throughout the brainstem, cortex, hippocampus, basal ganglia, and limbic structures.^2,3^ Synaptic zinc is loaded into glutamatergic vesicles by the zinc transporter protein zinc transporter 3 (ZnT3, *Slc30a3*).^1,2,4,5^ ZnT3 knockout (KO) mice, which lack synaptic zinc, display a range of cognitive and sensory deficits,^6–12^ and have behavioral deficits associated with autism^13^ and schizophrenia.^9,11^ Together, these findings strongly suggest that synaptic zinc signaling is important for sensory, motor, and cognitive processing.

Synaptic zinc acts as a neuromodulator that inhibits NMDA receptor (NMDAR) function,^14–19^ AMPA receptor (AMPAR) function,^20^ GABA_A_ receptor function,^21^ and triggers endocannabinoid synthesis.^22^ At different concentrations, exogenous zinc also inhibits and potentiates glycine receptor function,^23^ AMPAR function,^24–28^ and kainate receptor function.^27,29,30^ These findings suggest that synapse-specific levels of zinc—along with differences in postsynaptic factors, such as receptor subunit types and postsynaptic transporters—are an important factor in how synaptic zinc shapes synaptic transmission.^1,4,14,16,17,28,31–34^ Accordingly, determining the synapse-specific dynamics of synaptic zinc is crucial for our understanding of normal synaptic function and sensory processing.

Previous reports, at times, directly contradict one another about the effects of synaptic zinc signaling on synaptic transmission, particularly on AMPAR and NMDAR function, with divergent conclusions about how and if zinc affects these glutamate receptors during synaptic transmission.^14–18,20,22,35–37^ How these disparate findings map onto the natural roles of synaptic zinc signaling in the brain is not well understood, but the lack of agreement points to a larger gap in our understanding of how the range of effects of synaptic zinc support the normal function of neuronal circuits.

Here, to understand the roles of synaptic zinc signaling at specific types of glutamatergic synapses used for auditory processing, we used acute brain slices of the auditory cortex, and performed whole-cell patch-clamp recordings from two populations of layer 5 cortical neurons. We optogenetically stimulated distinct classes of presynaptic neurons and recorded the synaptic inputs to distinct classes of postsynaptic neurons. We also used *in vivo* 2-photon calcium imaging of the same neuronal populations in awake mice during sound processing. We identify roles for synaptically released zinc in differentially potentiating AMPAR function at specific glutamatergic cortical synapses which support normal auditory processing. Thus, synaptic zinc contributes to cell-type-specific sound processing by widening or sharpening the sound-frequency tuning bandwidths of specific neuronal populations in awake mice. Importantly, our results offer a unifying explanation for contradictory reports of the effects of synaptic zinc on synaptic transmission.

## RESULTS

### Synaptic zinc potentiates AMPAR function at synapses between IT-type neurons and PT-type neurons

**Figure 1.**
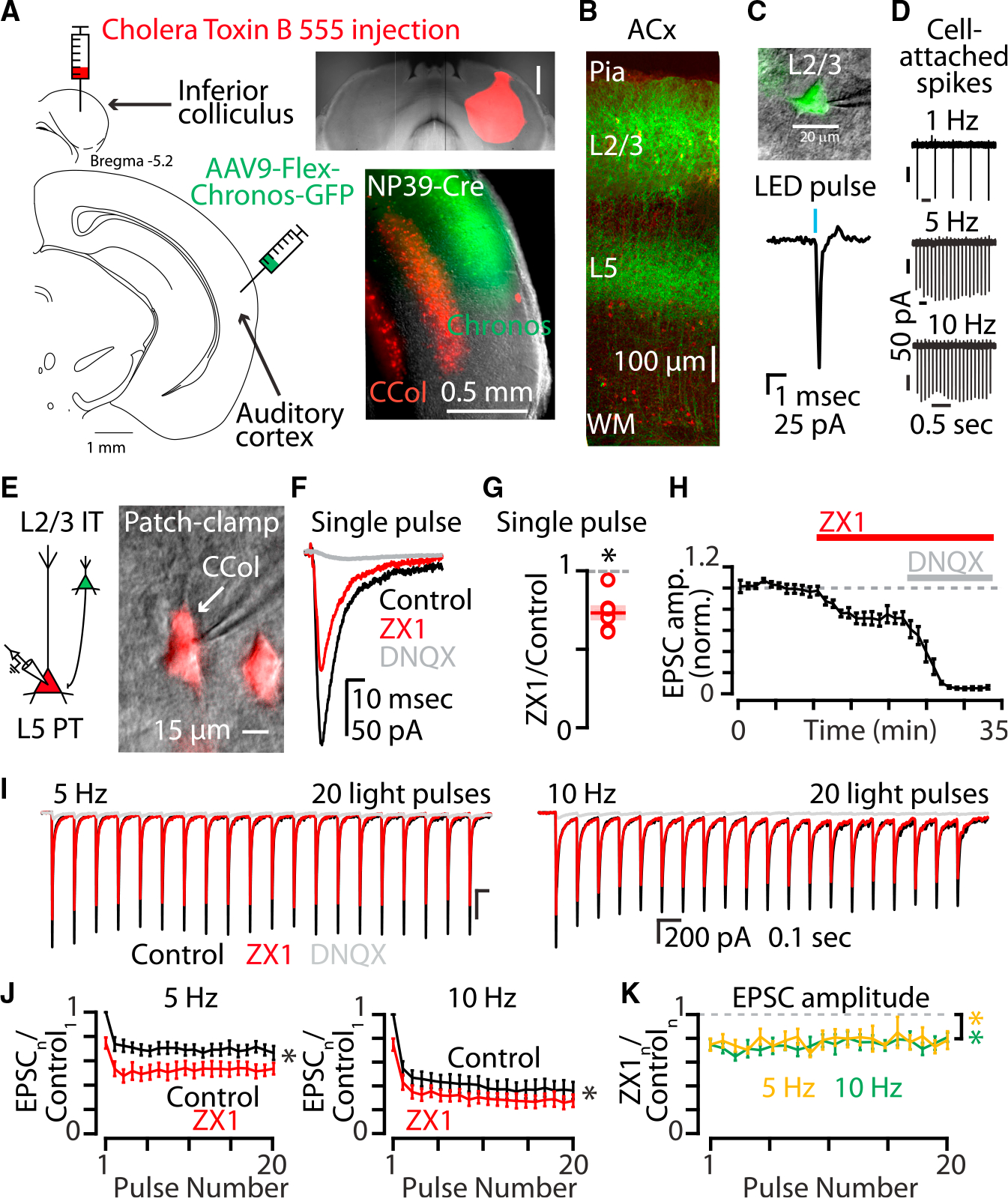
Synaptic zinc release enhances AMPAR function throughout trains of stimuli at synapses between layer 2/3 IT-type neurons and layer 5 PT-type neurons (A) Left: cartoon showing experimental injection scheme for labeling neurons in the auditory cortex. Right, top: example image of acute brain slice showing inferior colliculus injection with Cholera toxin subunit B conjugated to Alexa 555 fluorophore (CTB-555). Scale bar: 0.5 mm. Right, bottom: example image of acute brain slice of the right auditory cortex of an NP39-Cre mouse with corticocollicular neurons in layer 5 (red) and NP39-Cre neurons in layer 2/3 (green). Scale bar: 0.5 mm. (B) Two-photon image of acute brain slice of the auditory cortex with chronos-expressing NP39-Cre neurons with somata and proximal dendrites in layer 2/3 and axonal arborizations in layer 5. Scale bar: 100 μm. (C) Top: example image of cell-attached configuration patch-clamp recording of a chronos-expressing layer 2/3 neuron. Scale bar: 20 μm. Bottom: example LED pulse-evoked action potential recorded in cell-attached mode via the pipette. (D) Stimulus trains of LED pulses at 1, 5, and 10 Hz evoked action potentials in layer 2/3 chronos-expressing neurons. (E) Left: cartoon illustrating synaptic connection between layer 2/3 neuron expressing chronos (green) and a postsynaptic layer 5 neuron expressing CTB-555 (red). Right: example image of a PT-type corticocolliccular neuron and patch pipette in whole-cell patch-clamp configuration. Scale bar: 15 μm. (F) Example traces showing the average of AMPAR EPSCs in response to a single pulse of blue light. Black, control EPSC; red, EPSC after the addition of ZX1; gray, EPSC after the addition of DNQX. (G) Average effect of ZX1 on the amplitude of AMPAR EPSCs in response to a single pulse of light (p = 0.0019, n = 6 cells from four mice; paired t test). (H) Average time course of AMPAR EPSC amplitudes in response to a single pulse of light after the addition of ZX1 and the subsequent addition of DNQX. (I) Example averaged EPSCs from trains of light pulses at 5 Hz (left) and 10 Hz (right). Color scheme same as in (F). (J) Average effect of ZX1 (red) on the amplitude of averaged AMPAR EPSCs in response to trains of light pulses at 5 Hz (left) and 10 Hz (right), normalized to the average of the first control EPSC in the stimulus train (5 Hz: p = 0.0021, n = 6 cells from four mice; 10 Hz: p = 0.008, n = 6 cells from four mice; 2-way repeated-measures ANOVAs). (K) Average effect of ZX1 on the amplitude of AMPAR EPSCs in response to trains of light pulses at 5 Hz (yellow) and 10 Hz (green), normalized to the amplitude of each corresponding average control EPSC. (Compared with no change: 5 Hz: p = 0.0038, n = 6 cells from four mice; 10 Hz: p = 0.0023, n = 6 cells from four mice; 2-way repeated-measures ANOVAs.). See also Figure S1. Asterisks indicate significant p values. Data are represented as mean ± SEM.

To investigate the effects of synaptically released zinc at specific intracortical synaptic pathways, we first focused on a subset of intratelencephalic (IT)-type glutamatergic neurons in layer 2/3 of the mouse auditory cortex. These neurons have high levels of ZnT3 expression^38–40^ and contain high levels of vesicular zinc in presynaptic terminals.^41,42^ To target this specific population of neurons, we used the NP39 mouse line which selectively expresses Cre-recombinase in layer 2/3 IT-type neurons^43–45^ (Figures 1A and 1B). In order to selectively optogenetically activate these neurons, mice received a stereotaxic injection of an adeno-associated virus AAV9-hSyn1-FLEX-Chronos-GFP, which induces the expression of the fast excitatory opsin Chronos in a Cre-dependent manner.^46–48^ The same mice also received an injection of the retrograde tracer cholera toxin subunit B (CTB) Alexa 555^49^ in the right inferior colliculus to fluorescently label pyramidal tract (PT)-type corticocollicular neurons in layer 5 of the auditory cortex (Figure 1A), which receive strong interlaminar glutamatergic synaptic inputs from layer 2/3 IT-type neurons.^50^ Seven to 21 days after the injections, acute brain slices containing the auditory cortex were prepared (STAR Methods). We verified that blue light was able to evoke reliable action potential firing by chronos-expressing neurons (Figures 1C and 1D). To record light-evoked synaptic inputs to layer 5 corticocollicular neurons, fluorescently labeled corticocollicular neurons were visually targeted for whole-cell patch-clamp recordings (Figure 1E). Blue light pulses elicited excitatory postsynaptic currents (EPSCs) in corticocollicular neurons that were mediated by AMPARs (Figures 1F–1H). We observed more short-term depression at 10 Hz vs. 5 Hz (Figure 1J), consistent with enhanced depression at higher stimulus frequencies which occurs at cortical synapses.^51,52^ To quantify the effects of synaptic zinc on these EPSCs, we bath applied 100 μM of the fast, extracellular zinc chelator ZX1.^14,16,18,20,21,35^ ZX1 application resulted in a reduction of ~25% in the amplitude of the first AMPAR EPSC (Figures 1F–1H), suggesting that synaptic zinc release serves to potentiate AMPAR EPSCs between IT-type layer 2/3 neurons and layer 5 corticocollicular neurons. We next delivered trains of light pulses and found that ZX1 also caused a significant reduction in AMPAR EPSC amplitudes throughout the stimulus train (Figures 1I and 1J). Together these results suggest that synaptically released zinc serves to potentiate synaptic inputs from IT-type layer 2/3 neurons to layer 5 corticocollicular neurons. ZX1 did not change the relative steady state of the EPSC amplitudes reached during the train (Figures S1A and S1B), suggesting that zinc does not affect the relative magnitude of the EPSCs within the train, but rather scales the EPSCs throughout the train (Figure 1K). Consistent with a specific postsynaptic effect of ZX1 on AMPAR EPSCs, paired-pulse ratios were not affected by the addition of ZX1 (Figures S1C and S1D), suggesting that ZX1 does not affect presynaptic release properties at these synapses. Further supporting a postsynaptic locus of the effects of ZX1, light-evoked spike fidelity of chronos-expressing neurons was not affected by the addition of ZX1 (Figures S1G and S1H), suggesting that the reduction in AMPAR EPSC amplitude is not due to reduced photoexcitability of the presynaptic neurons. Together, these results suggest synaptic zinc released from layer 2/3 IT-type neurons potentiates the strength of their AMPAR-mediated inputs onto layer 5 corticocollicular neurons.

**Figure 2.**
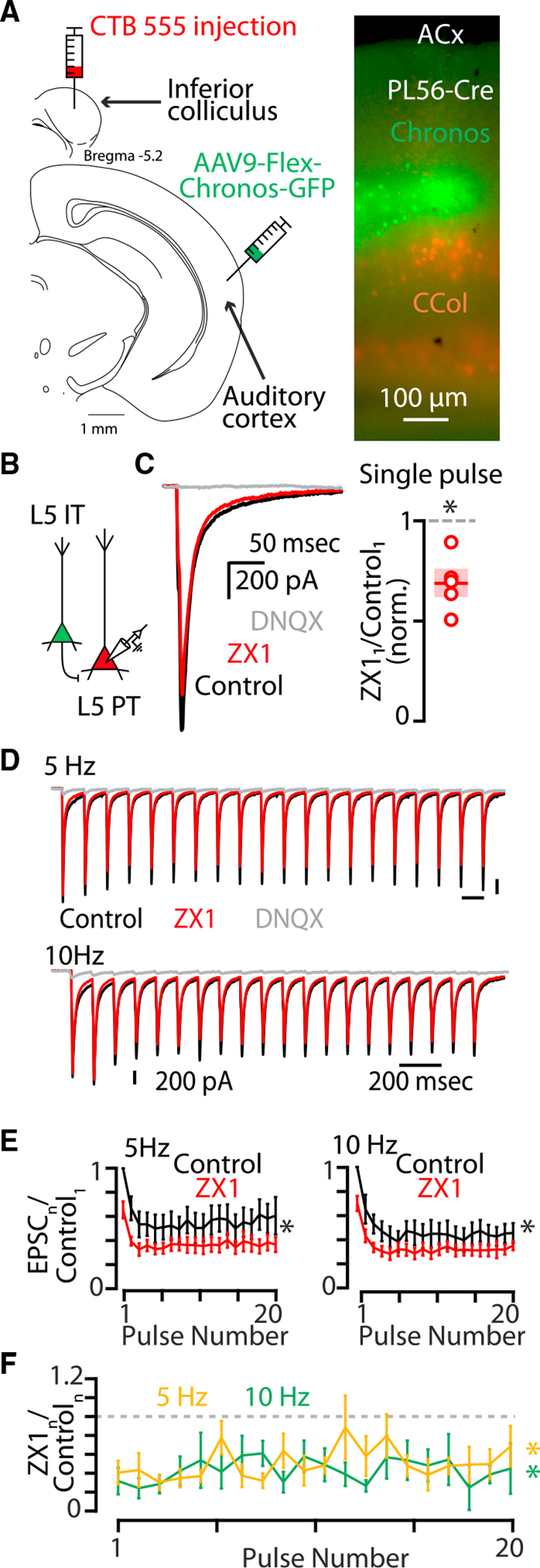
Synaptic zinc release enhances AMPAR function at synapses between layer 5 IT-type neurons and layer 5 PT-type neurons (A) Left: cartoon showing experimental injection scheme for labeling neurons in the auditory cortex. Right: example image of acute brain slice of the right auditory cortex of a PL56-Cre mouse with corticocollicular neurons in layer 5 (red) and PL56-Cre neurons in layer 5 (green). Scale bar: 100 μm. (B) Cartoon illustrating presynaptic layer 2/3 neuron expressing chronos (green) and a postsynaptic layer 5 neuron expressing CTB-555 (red). (C) Left: example traces showing average AMPAR EPSCs in response to a single pulse of blue light. Black, control EPSC; red, EPSC after the addition of ZX1; gray, EPSC after the addition of DNQX. Right: average effect of ZX1 on the amplitude of AMPAR EPSCs in response to a single pulse of light (p = 0.0080, n = 5 cells from four mice, paired t test). (D) Example averaged EPSCs from trains of light pulses at 5 Hz (top) and 10 Hz (bottom). Color scheme same as in (C). (E) Average effect of ZX1 (red) on the average amplitude of AMPAR EPSCs amplitude in response to trains of light pulses at 5 Hz (left) and 10 Hz (right), normalized to the average of the first control EPSC in the stimulus train (5 Hz: p = 0.0359, n = 5 cells from four mice; 10 Hz: p = 0.0339, n = 5 cells from four mice; 2-way repeated-measures ANOVA). Color scheme same as in (C). (F) Average effect of ZX1 on the amplitude of AMPAR EPSCs in response to trains of light pulses at 5 Hz (yellow) and 10 Hz (green) normalized to the average amplitude of control EPSCs (5 Hz: p = 0.0184, n = 5 cells from four mice; 10 Hz: p = 0.0055, n = 5 cells from four mice; 2-way repeated-measures ANOVA). See also Figure S1. Asterisks indicate significant p values. Data are represented as mean ± SEM. See Table S1 for detailed statistics.

The enhancing effects of synaptic zinc on AMPAR function could be unique to layer 2/3 IT-type inputs to layer 5 corticocollicular neurons, or they could be a general feature of IT-type presynaptic neurons. To investigate whether the same effects of synaptic zinc occur from a different set of IT-type neurons, we recorded synaptic inputs from layer 5 IT-type neurons in acute brain slices, another source of intracortical glutamatergic input to layer 5 PT-type neurons.^53^ In experiments similar to those in Figure 1, PL56-Cre mice^43,54,55^ were used to express chronos in layer 5 IT-type neurons (Figure 2A). Zinc signaling had a similar potentiating effect on AMPAR EPSCs between layer 5 IT-type neurons and corticocollicular neurons (Figures 2B–2F and S1C) suggesting that synaptic zinc acts in a similar fashion to potentiate AMPAR function at both layer 2/3 IT-PT and layer 5 IT-PT synapses in auditory cortex.

**Figure 3.**
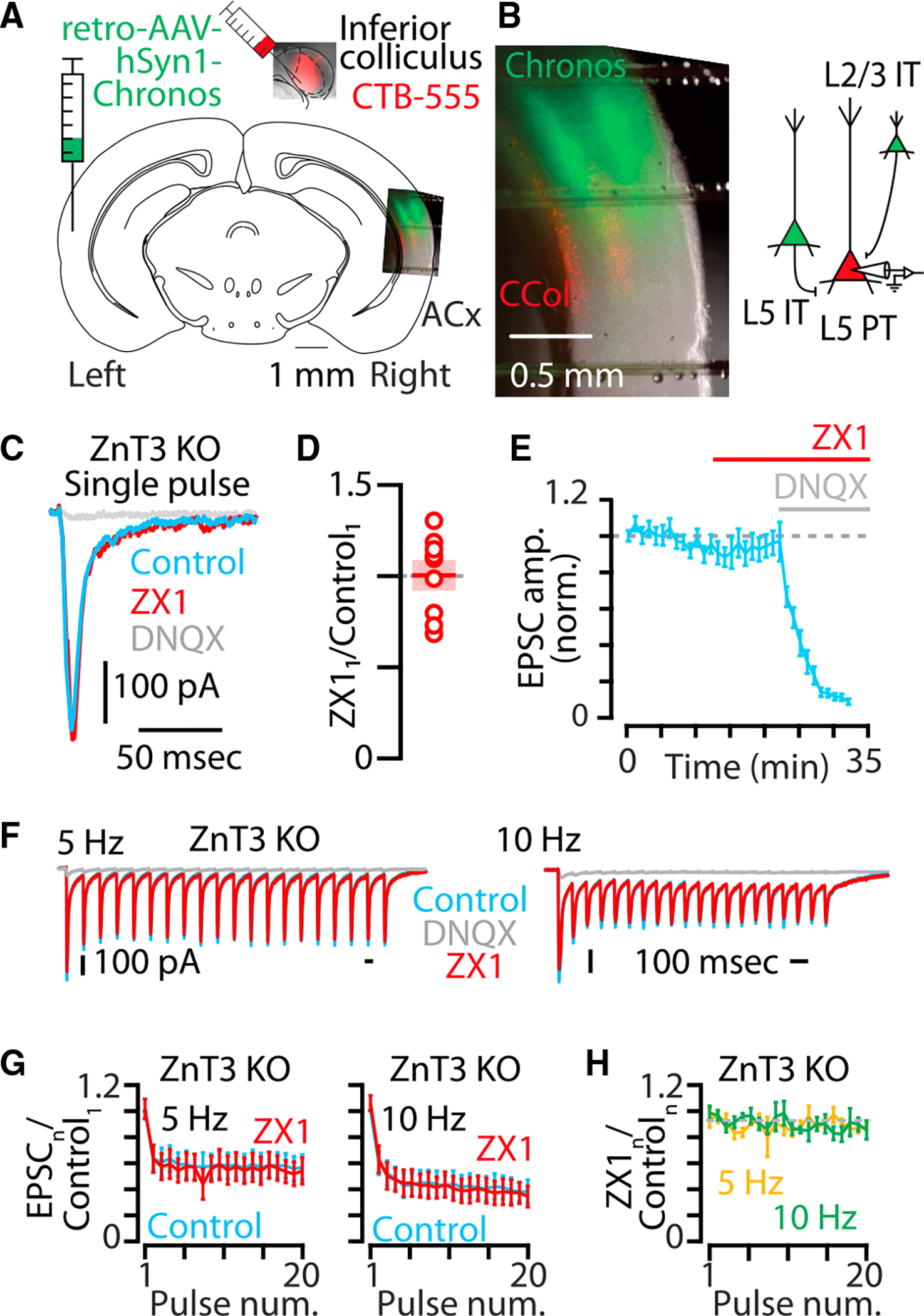
ZnT3-dependent, synaptically released zinc enhances AMPAR function at synapses between IT-type callosal neurons and layer 5 PT-type neurons (A) Cartoon showing experimental injection scheme for labeling neurons in the auditory cortex in ZnT3 KO mice. (B) Left: example image of acute brain slice of the right auditory cortex of a ZnT3 KO mouse with corticocollicular neurons in layer 5 (red) and IT-type callosal neurons (green). Right: cartoon illustrating presynaptic IT-type neurons expressing chronos (green) and a postsynaptic layer 5 neuron expressing CTB-555 (red). Scale bar: 0.5 mm. (C) Example traces showing averaged AMPAR EPSCs in response to a single pulse of blue light in ZnT3 KO. Blue, control EPSC; red, EPSC after the addition of ZX1; gray, EPSC after the addition of DNQX. (D) Average effect of ZX1 on the amplitude of AMPAR EPSCs in response to a single pulse of light (p = 0.9549, n = 9 cells from seven mice, paired t test). (E) Average time course of AMPAR EPSC amplitudes in response to a single pulse of light after the addition of ZX1 and the subsequent addition of DNQX. (F) Example averaged EPSCs from trains of light pulses at 5 Hz (left) and 10 Hz (right). Color scheme same as in (C). (G) Average effect of ZX1 (red) on the amplitude of AMPAR EPSCs amplitude in response to trains of light pulses at 5 Hz (left) and 10 Hz (right), normalized to the average of the first control EPSC in the stimulus train (5 Hz: p = 0.56, n = 9 cells from seven mice; 10 Hz: p = 0.9558, n = 9 cells from seven mice; 2-way repeated-measures ANOVA). Color scheme same as in (C). (H) Average effect of ZX1 on the amplitude of AMPAR EPSCs normalized to the amplitude of each corresponding control EPSC in response to trains of light pulses at 5 Hz (yellow) and 10 Hz (green) (5 Hz: p = 0.4941, n = 9 cells from seven mice; 10 Hz: p = 0.9123, n = 9 cells from seven mice; 2-way repeated-measures ANOVA). Asterisks indicate significant p values. Data are represented as mean ± SEM. See Table S1 for detailed statistics.

**Figure 4.**
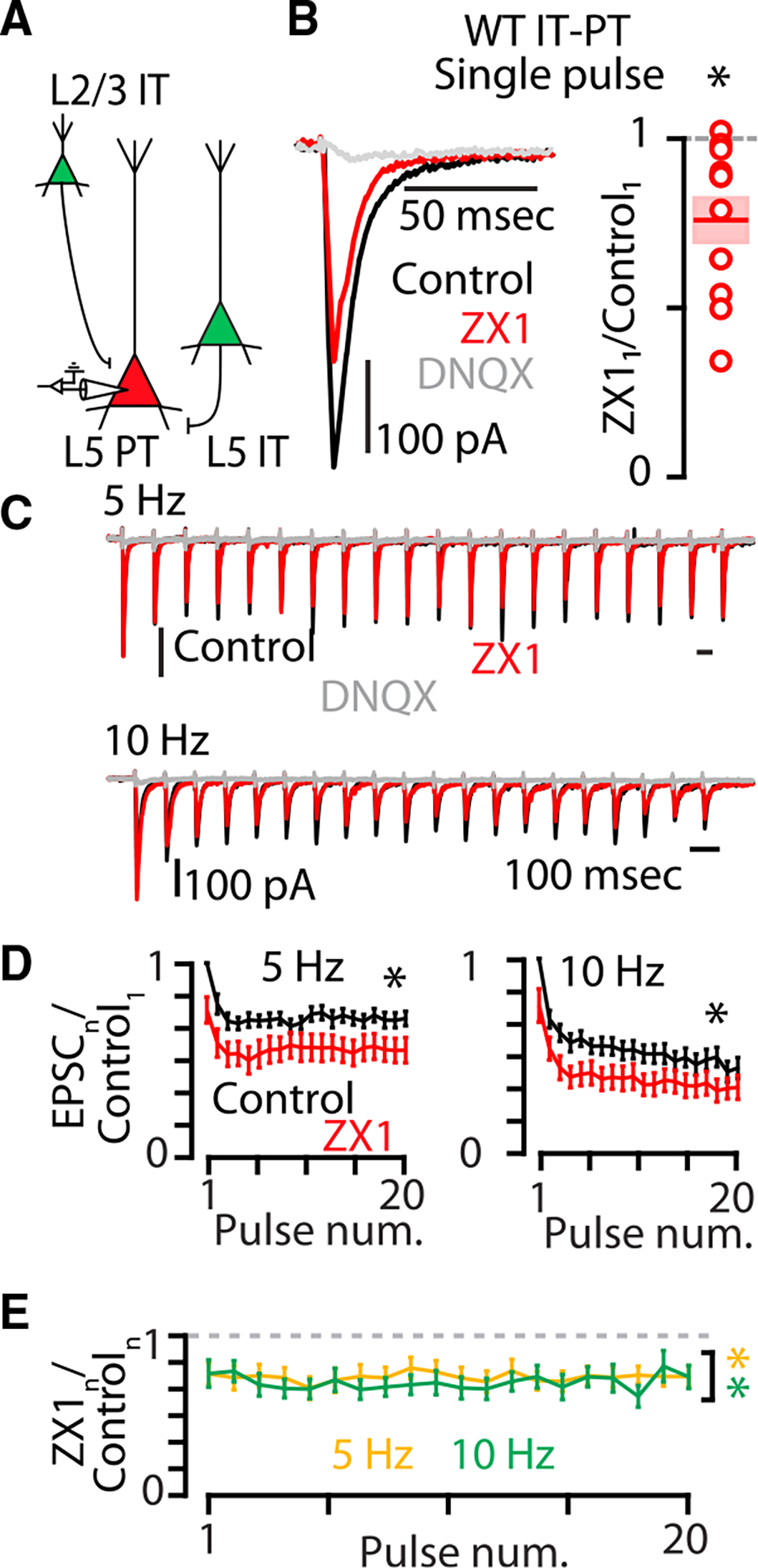
Synaptic zinc released from IT-type corticocallosal neurons in WT mice enhances AMPAR function at IT-PT synapses (A) Cartoon illustrating presynaptic IT-type neurons expressing chronos (green) and a postsynaptic layer 5 neuron expressing CTB-555 (red). (B) Left: example traces showing averaged AMPAR EPSCs in response to a single pulse of blue light in WT mice. Black, control EPSC; red, EPSC after the addition of ZX1; gray, EPSC after the addition of DNQX. Right: average effect of ZX1 on the amplitude of AMPAR EPSCs in response to a single pulse of light (p = 0.0031, n = 12 cells from ten mice, paired t test). (C) Example averaged EPSCs from trains of light pulses at 5 Hz (top) and 10 Hz (bottom). Black, control EPSC; red, EPSC after the addition of the zinc chelator ZX1; gray, EPSC after the addition of DNQX. Color scheme same as in (B). (D) Average effect of ZX1 (red) on the amplitude of AMPAR EPSCs amplitude in response to trains of light pulses at 5 Hz (left) and 10 Hz (right), normalized to the average of the first control EPSC in the stimulus train (5 Hz: p = 0.0015, n = 12 cells from ten mice; 10 Hz: p = 0.0001, n = 12 cells from ten mice; 2-way repeated-measures ANOVAs). Color scheme same as in (B). (E) Average effect of ZX1 on the amplitude of AMPAR EPSCs normalized to the amplitude of each corresponding control EPSC in response to trains of light pulses at 5 Hz (yellow) and 10 Hz (green) (5 Hz: p = 0.0014, n = 12 cells from ten mice; 10 Hz: p = 0.0004, n = 12 cells from ten mice; 2-way repeated-measures ANOVAs). See also Figure S1. Asterisks indicate significant p values. Data are represented as mean ± SEM. See Table S1 for detailed statistics.

To confirm that synaptic zinc is responsible for the effects of zinc on AMPAR-mediated synaptic signaling, we performed similar experiments as above in ZnT3 KO mice, which lack ZnT3 and synaptic zinc.^2^ To induce the expression of chronos in IT-type neurons, we injected retro-AAV-hSyn1-GFP-Chronos into the left auditory cortex to retrogradely transfect callosally projecting IT-type neurons in both layer 2/3 and layer 5 of the right auditory cortex in ZnT3 KO mice (Figures 3A and 3B). Photoactivation of this group of IT-type neurons resulted in AMPAR EPSCs in layer 5 corticocollicular neurons (Figure 3C). In ZnT3 KO mice, ZX1 had no effect on AMPAR EPSCs (Figures 3C–3H) confirming that the effects of ZX1 observed at IT-PT synapses are due to the chelation of ZnT3-dependent synaptic zinc. To confirm that synaptic zinc released from this mixed presynaptic population of layer 2/3 and layer 5 IT-type neurons (Figure 4A) potentiates AMPAR EPSCs, we repeated this experiment in wild-type (WT) mice. ZX1 resulted in reductions in AMPAR EPSCs for the first EPSC (Figure 4B) and throughout the stimulus train (Figures 4C–4E), showing that the potentiation of AMPAR function by synaptic zinc occurs at these IT-PT synapses. Consistent with the effects of ZX1 having a postsynaptic locus at these synapses, ZX1 did not affect paired-pulse ratios (Figure S1D). Additionally, although synaptic zinc served to inhibit NMDAR EPSCs at IT-PT synapses at the first stimulus (Figures S2B and S2C) and along the stimulus train (Figures S2D and S2E), consistent with the well-established inhibitory effects of zinc on NMDAR function,^16–18,37,56–61^ the effects of synaptically released zinc on AMPAR EPSCs did not depend on NMDAR function because we observed the same potentiating effect of zinc on AMPAR EPSCs in the presence of the NMDAR-specific blocker AP5 (Figures S3A–S3F). Because copper can inhibit AMPAR currents,^62,63^ we tested for potential effects of copper on AMPAR EPSCs. The addition of the extracellular copper chelator bathocuproine sulfonate (BCS) had no effect on AMPAR EPSCs (Figure S4), and the subsequent addition of ZX1 resulted in significantly smaller AMPAR EPSC amplitudes in the presence of BCS (Figure S4). Taken together, these results show that ZnT3-dependent synaptic zinc potentiates AMPAR function.

### AMPAR potentiation by synaptic zinc is synapse specific

**Figure 5.**
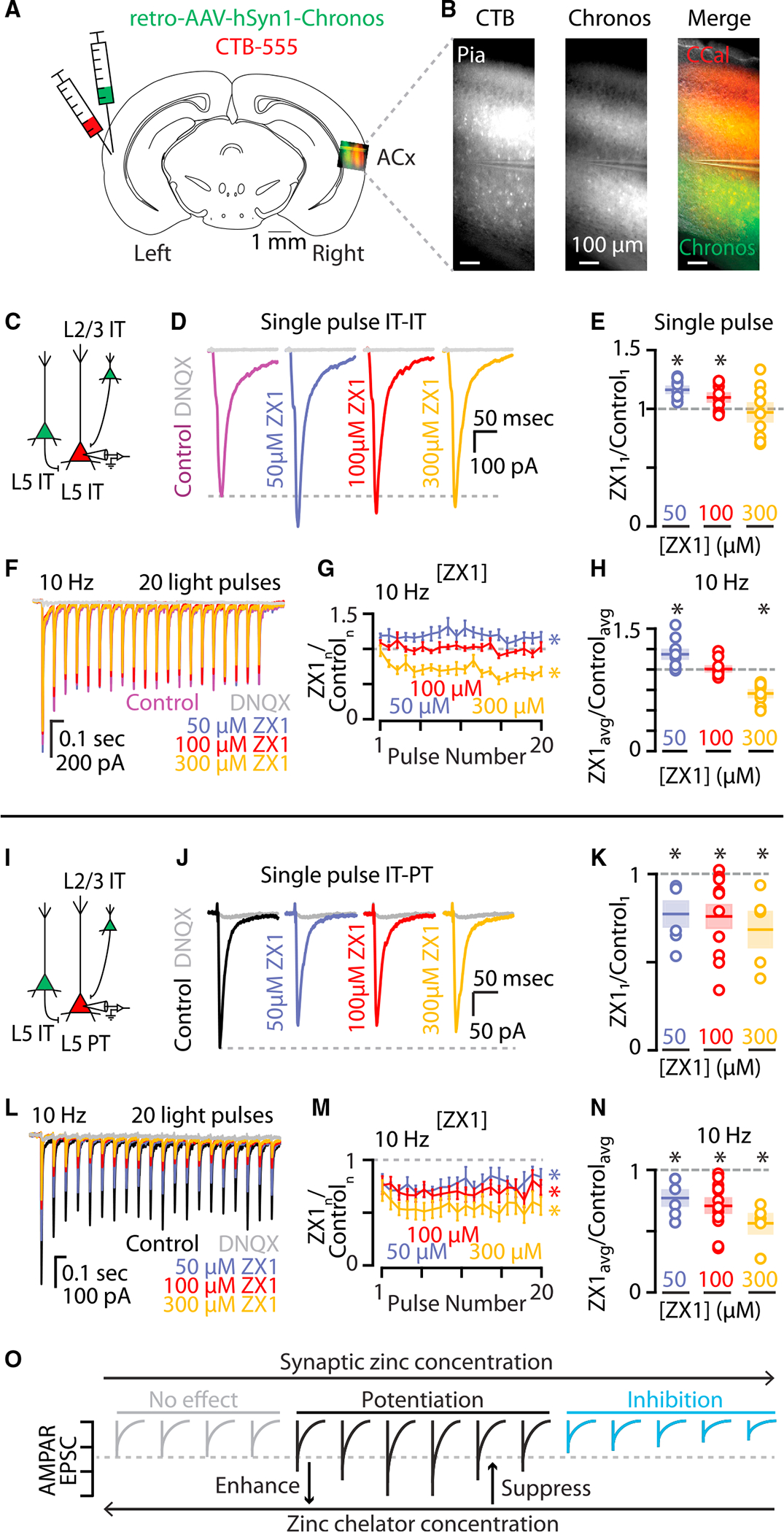
Synaptic zinc differentially potentiates IT-IT and IT-PT synapses in the auditory cortex (A) Cartoon showing experimental injection scheme for labeling neurons in the auditory cortex. (B) Example image of acute brain slice of the right auditory cortex of WT mouse with corticocallosal neurons in labeled with CTB (red) and chronos (green). Scale bar: 100 μm. (C) Cartoon illustrating presynaptic IT-type neurons expressing chronos (green) and a postsynaptic layer 5 IT-type neuron expressing only CTB-555 (red) and not chronos. (D) Example traces showing average IT-IT AMPAR EPSCs in response to a single pulse of blue light. Magenta, control EPSC; purple, EPSC after the addition of 50 μM ZX1; red, EPSC after the addition of 100 μM ZX1; gold, EPSC after the addition of 300 μM ZX1; gray, EPSC after the addition of DNQX. (E) Average effect of ZX1 on the average amplitude of IT-IT AMPAR EPSCs in response to a single pulse of light (50 μM ZX1: p = 0.00023822, n = 11 cells from nine mice; 100 μM ZX1: p = 0.0182, n = 11 cells from ten mice; 300 μM ZX1: p = 0.6962, n = 9 cells from nine mice; paired t test). Color scheme same as in (D). (F) Example average EPSCs from trains of light pulses at 10 Hz. Color scheme same as (D). (G) Average effect of ZX1 on the average amplitude of IT-IT AMPAR EPSCs normalized to the corresponding average control EPSC amplitudes in response to trains of light pulses at 10 Hz (10 Hz: 50 μM ZX1: p = 0.0054, n = 11 cells from nine mice; 100 μM ZX1: p = 0.8329, n = 11 cells from ten mice; 300 μM ZX1: p = 0.00010071, n = 9 cells from nine mice; 2-way repeated-measures ANOVA). Color scheme same as (D). (H) Average effect of ZX1 on the average amplitude of IT-IT AMPAR EPSCs in response to a stimulus train of blue light at 10 Hz normalized to the corresponding average control EPSC along the train at each concentration of ZX1 (50 μM ZX1: p = 0.0054, n = 11 cells from nine mice; 100 μM ZX1: p = 0.8329, n = 11 cells from ten mice; 300 μM ZX1: p = 0.00010071, n = 9 cells from nine mice; paired t test). Color scheme same as (D). (I) Cartoon illustrating presynaptic IT-type neurons expressing chronos (green) and a postsynaptic layer 5 PT-type neuron expressing CTB-555 (red). (J) Example traces at IT-PT synapses showing average AMPAR EPSCs in response to a single pulse of blue light. Black, control EPSC; purple, EPSC after the addition of 50 μM ZX1; red, EPSC after the addition of 100 μM ZX1; gold, EPSC after the addition of 300 μM ZX1; gray, EPSC after the addition of DNQX. (K) Average effect of ZX1 on the average amplitude of IT-PT AMPAR EPSCs in response to a single pulse of light (50 μM ZX1: p = 0.024, n = 6 cells from six mice; 100 μM ZX1: p = 0.0031, n = 12 cells from ten mice; 300 μM ZX1: p = 0.0334, n = 5 cells from five mice; paired t test). Data points for 100 μM ZX1 are also reported in Figure 4B. Color scheme same as in (J). (L) Example IT-PT EPSCs from trains of light pulses at 10 Hz. Color scheme same as in (J). (M) Average effect of ZX1 on the average amplitude of AMPAR EPSCs at IT-PT synapses normalized to the corresponding control EPSC amplitudes in response to trains of light pulses at 10 Hz (10 Hz: 50 μM ZX1: p = 0.0132, n = 6 cells from six mice; 100 μM ZX1: p = 0.0003627, n = 12 cells from ten mice; 300 μM ZX1: p = 0.0044, n = 5 cells from five mice; 2-way repeated-measures ANOVA). Color scheme same as (J). (N) Average effect of ZX1 on the average amplitude of IT-PT AMPAR EPSCs in response to a stimulus train of blue light normalized to the corresponding control EPSC along the train at each concentration of ZX1 (50 μM ZX1: p = 0.0132, n = 6 cells from six mice; 100 μM ZX1: p = 0.0003627, n = 12 cells from 10 mice; 300 μM ZX1: p = 0.0044, n = 5 cells from five mice; paired t test). Color scheme same as (J). (O) Cartoon of hypothesized relationship between synaptic zinc concentration and AMPAR EPSC amplitudes. Three concentration-dependent synaptic zinc regimes are proposed: a no effect regime of synaptic zinc concentration (gray) where synaptic zinc is not able to modulate AMPAR EPSCs, a potentiating regime of synaptic zinc concentration (black) where AMPAR EPSCs are potentiated by synaptic zinc, and an inhibitory regime of synaptic zinc concentration (blue) where AMPAR EPSCs are inhibited by synaptic zinc. Therefore, the enhancing or suppressing effects of zinc chelation on AMPAR EPSCs depend on the synapse-specific concentration of zinc and the concentration of zinc chelator used. See also Figure S5. Data from Figures 4B and 4G are used in (K) and (M). Asterisks indicate significant p values. Data are represented as mean ± SEM. See Table S1 for detailed statistics.

The ability of synaptically released zinc to potentiate AMPAR EPSCs could be a feature of all IT-type presynaptic neurons or due to a specific combination of pre- and postsynaptic cortical neurons. The intracortical glutamatergic pathways from IT-type neurons in layer 2/3 and layer 5 also provides synaptic input to layer 5 IT-type neurons, which have long-range axonal projections throughout the telencephalon.^49,50,64–72^ Therefore, by recording IT-type inputs to layer 5 IT-type neurons, we can stimulate the same population of IT-type neurons as above but record synaptic inputs to a different class of postsynaptic neurons (Figures 5A–5C). At these IT-IT synapses, we found that 100 μM ZX1 had very different effects on AMPAR EPSCs: an increase in the first EPSC amplitude (Figures 5D and 5E) followed by no significant changes in subsequent EPSCs (Figures 5F–5H). This is a stark contrast to the robust reductions of AMPAR EPSCs by 100 μM ZX1 at IT-PT synapses (Figures 5I–5N and 4). Multiple scenarios could explain these results, including (1) that zinc levels are high enough to inhibit AMPAR EPSCs at IT-IT synapses^25,73^ or (2) that zinc levels are in a different range of potentiating effects of zinc on AMPAR EPSCs at IT-IT synapses.^26^ We hypothesized that increasing the concentration of ZX1 would differentiate between zinc inhibition vs. zinc potentiation (Figure 5O). If zinc levels are high enough to inhibit AMPAR EPSCs, then increased ZX1 should only result in increased amplitudes of AMPAR EPSCs; however if zinc levels are potentiating, then increased ZX1 could also cause no change or smaller AMPAR EPSCs (Figure 5O). Consistent with the hypothesis of a potentiating amount of zinc that is reduced by zinc chelation at IT-IT synapses, higher levels of ZX1 (300 μM ZX1) resulted in no change in the first EPSC (Figures 5D and 5E) and decreased AMPAR EPSC amplitudes throughout the train (Figures 5F–5H). This result suggests that synaptic zinc is not inhibiting AMPAR function, but rather that increased zinc chelation shifts the effects of the remaining synaptic zinc to be less potentiating of AMPAR EPSCs (Figure 5O; Figures S5A–S5C). Because zinc has concentration-dependent, bimodal effects on AMPARs,^25,26,74^ if AMPAR EPSCs can also become larger with lower amounts of zinc chelation, this suggests that synaptic zinc levels are sufficient to potentiate, but not inhibit, AMPAR function at IT-IT synapses (Figure 5O). Consistent with a range of potentiating effects of synaptic zinc, 50 μM ZX1 resulted in larger AMPAR EPSC amplitudes (Figures 5E–5H and S5A–S5C). This result suggests that decreased zinc chelation shifts the effects of the remaining zinc to be more potentiating of AMPAR EPSCs (Figures 5G, 5H, and S5A–S5C). The copper chelator BCS did not affect AMPAR EPSC amplitudes at IT-IT synapses (Figure S4), further supporting the conclusion that synaptic zinc is potentiating AMPAR EPSCs at these synapses. The enhancement or suppression of the potentiating effects on AMPAR EPSCs were specific to IT-IT synapses because different levels of ZX1 at IT-PT synapses did not reveal bidirectional effects of zinc on AMPAR function (Figures 5J–5N and S5E–S5G). We did not observe differences in the AMPAR EPSC decay kinetics between IT-IT or IT-PT synapses in control or after ZX1 (Figures S5I and S5J). Paired-pulse ratios were not affected by any of the concentrations of ZX1 applied at either IT-IT or IT-PT synapses (Figures S5D and S5H), consistent with a postsynaptic effect of synaptic zinc. The relative magnitude of the steady-state EPSCs amplitudes at 5 Hz and 10 Hz stimulation was not affected by the concentration of ZX1 used (Figures S6A–S6D), suggesting that synaptic zinc does not have activity-dependent effects on steady-state depression at these stimulation frequencies at IT-IT or IT-PT synapses.

The potentiation of AMPAR function by zinc is hypothesized to occur by decreasing rapid glutamate desensitization of the receptors because the potentiating effects of exogenous zinc can be occluded by cyclothiazide, an AMPAR agonist that potentiates AMPAR function (Figures S7A and S7B) and reduces glutamate desensitization.^25,28,46,73,75^ The addition of cyclothiazide slowed the decay kinetics (Figures S7A) and occluded the effects of both high (300 μM) and low (50 μM) ZX1 on AMPAR EPSCs (Figures S7C and S7D), without unmasking any inhibitory effects of zinc on AMPAR function. These findings support the conclusion that synaptic zinc functions within a potentiating concentration at IT-IT synapses. Because different amounts of ZX1 have different effects at IT-IT synapses, but the same effects at IT-PT synapses, these results suggest that zinc in the cleft at IT-IT synapses reaches higher concentrations than at IT-PT synapses, but that at both types of synapses, synaptically released zinc serves to potentiate AMPAR function, in a synapse-specific manner (Figures 5O and 5P).

### Synaptic zinc differentially modulates the sound-frequency tuning bandwidths of IT- and PT-type neurons in awake mice

**Figure 6.**
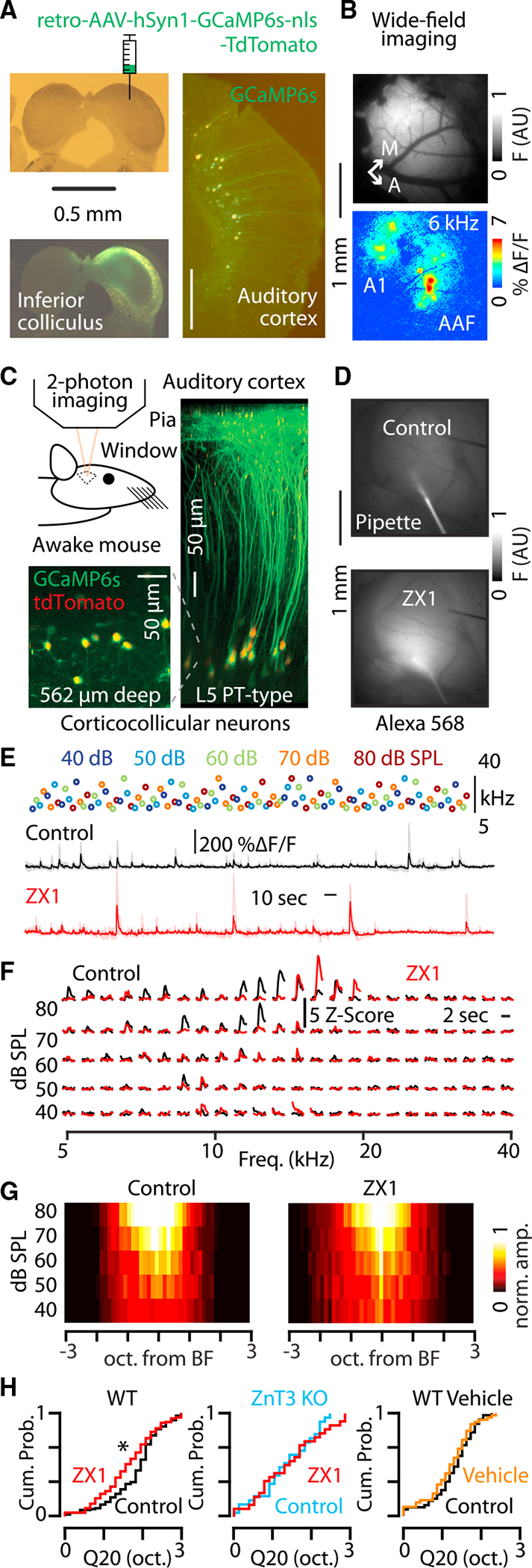
Synaptic zinc widens the sound-frequency tuning bandwidth of PT-type neurons in awake mice (A) Top left: bright-field image of brain slice containing the inferior colliculus. Cartoon shows injection of AAVrg-hSyn1-GCaMP6s-nls-tdTomato in the right hemisphere of the inferior colliculus. Scale bar: 0.5 mm. Bottom left: fluorescent image of the slice above showing GCaMP6s expression in the inferior colliculus. Scale bar: 0.5 mm. Right: fluorescent image of the auditory cortex in the same animal showing GCaMP6s expression in layer 5 corticocollicular neurons. Scale bar: 0.5 mm. (B) Top: image of GCaMP6s fluorescence from cortical surface through a craniotomy over the auditory cortex. Bottom: image of the changes in GCaMP6s fluorescence in response to a 50 dB SPL 6 kHz tone presented to the animal. A1, primary auditory cortex; AAF, anterior auditory field. Scale bar: 1 mm. (C) Top left: cartoon showing *in vivo* 2-photon calcium imaging in awake mice. Bottom left: example 2-photon fluorescent image of somata of corticocollicular neurons in layer 5 of the auditory cortex in an awake mouse. Green, GCaMP6s; red, tdTomato. Right: three-dimensional projection of corticocollicular neurons created from a series of 2-photon imaging planes taken from the pia to layer 5 in the auditory cortex of an awake mouse. Scale bar: 50 μm. (D) Top: fluorescent image of pipette containing ZX1 and Alexa 568 inserted into the cortex in control. Bottom: fluorescent image of the same field of view after infusing the cortex with ZX1 and Alexa 568. Scale bar: 1 mm. (E) Top: schematic of the sound presentation block used to measure sound-frequency tuning bandwidth of neurons in awake mice. Each circle represents one sound presentation; color indicates sound level; vertical position indicates sound frequency; the stimulus block contained 125 unique combinations of sound level and sound frequency. Middle: GCaMP6s-mediated fluorescent response of an individual neuron to the sound presentation block above in control (black). Bottom: the response of the same neuron to the same stimulus presentation block after the infusion of ZX1 into the brain. Traces are aligned in time with each other and with the sound presentation block. (F) Example responses to the sound frequency presentation block from a single neuron in E in control (black) and in ZX1 (red). (G) Group data showing the population sound-evoked responses in control(left) and in ZX1 (right). (H) Left: cumulative probability distribution showing the effect of ZX1 (red) on the Q20 of corticocollicular neurons (ZX1 vs. control [black], p = 0.002, n = 115 neurons from eighteen mice; signed-rank test). Middle: cumulative probability distribution showing the effect of ZX1 on the Q20 of corticocollicular neurons in ZnT3 KO mice (ZX1 [red] vs. ZnT3 KO control [blue], p = 0.33, n = 49 neurons from six mice; signed-rank test). Right: cumulative probability distribution showing the effect of vehicle infusions on the Q20 of corticocollicular neurons in WT mice (control [black] vs. vehicle [orange], p = 0.07, n = 49 neurons from nine mice; signed-rank test). Asterisks indicate significant p values. Data are represented as mean ± SEM. See Table S1 for detailed statistics.

Thus far, our findings of AMPAR function potentiation have been based on optogenetic activation of specific neurons in acute brain slices, which are not naturally occurring stimuli. Therefore, we hypothesized that the effects of synaptically released zinc would support neuronal processing in awake animals in response to sound. To test this hypothesis, we performed calcium imaging of the same neuronal populations in awake mice during sound processing. To assess the effects of synaptically released zinc on corticocollicular neuron activity *in vivo*, we first injected retro-AAV-hSyn1-jGCaMP7b^76^ or retro-AAV-hSyn1-GCaMP6s^77^ into the inferior colliculus to induce the expression of GCaMP7b or 6s in corticocollicular neurons in the auditory cortex via transfection of their axon terminals in the inferior colliculus (Figure 6A). We then targeted corticocollicular neurons in the primary auditory cortex, which we located in each mouse by mapping the tonotopic wide-field calcium response areas using low-frequency, low-intensity sounds^11,12,21,78–83^ (Figure 6B). Following this wide-field mapping, we switched to high-resolution 2-photon imaging of corticocollicular neuron somata in layer 5 in the same animal (Figure 6C) to measure the sound-frequency tuning bandwidth of multiple neurons in each mouse by presenting a range of pure-tone sounds from 40 to 80 dB SPL spanning 5–40 kHz in 1/8 octave steps (Figure 6E). After measuring sound-evoked responses, we infused 100 μM ZX1 into the brain via a pipette inserted into the cortex adjacent to the primary auditory cortex (Figure 6D; STAR Methods).^11,12,21,80^ We then quantified the effect of zinc signaling on sound-evoked responses by remeasuring neuronal responses to the same sounds with reduced levels of zinc. Consistent with an enhancing effect of synaptic zinc at IT-PT synapses observed in our *ex vivo* experiments above, zinc chelation resulted in a sharpening of the sound-frequency tuning bandwidth of corticocollicular neurons *in vivo* (Figures 6E–6G). These effects were due to ZnT3-dependent synaptic zinc because ZX1 infusions in ZnT3 KO mice had no effect on the sound-frequency tuning bandwidth of corticocollicular neurons (Figure 6H). Further confirming the specific role for synaptic zinc in widening the sound-frequency tuning bandwidth of corticocollicular neurons, infusions of artificial cerebrospinal fluid (vehicle) also had no effect on the sound-frequency tuning bandwidth of corticocollicular neurons in WT mice (Figure 6H).

**Figure 7.**
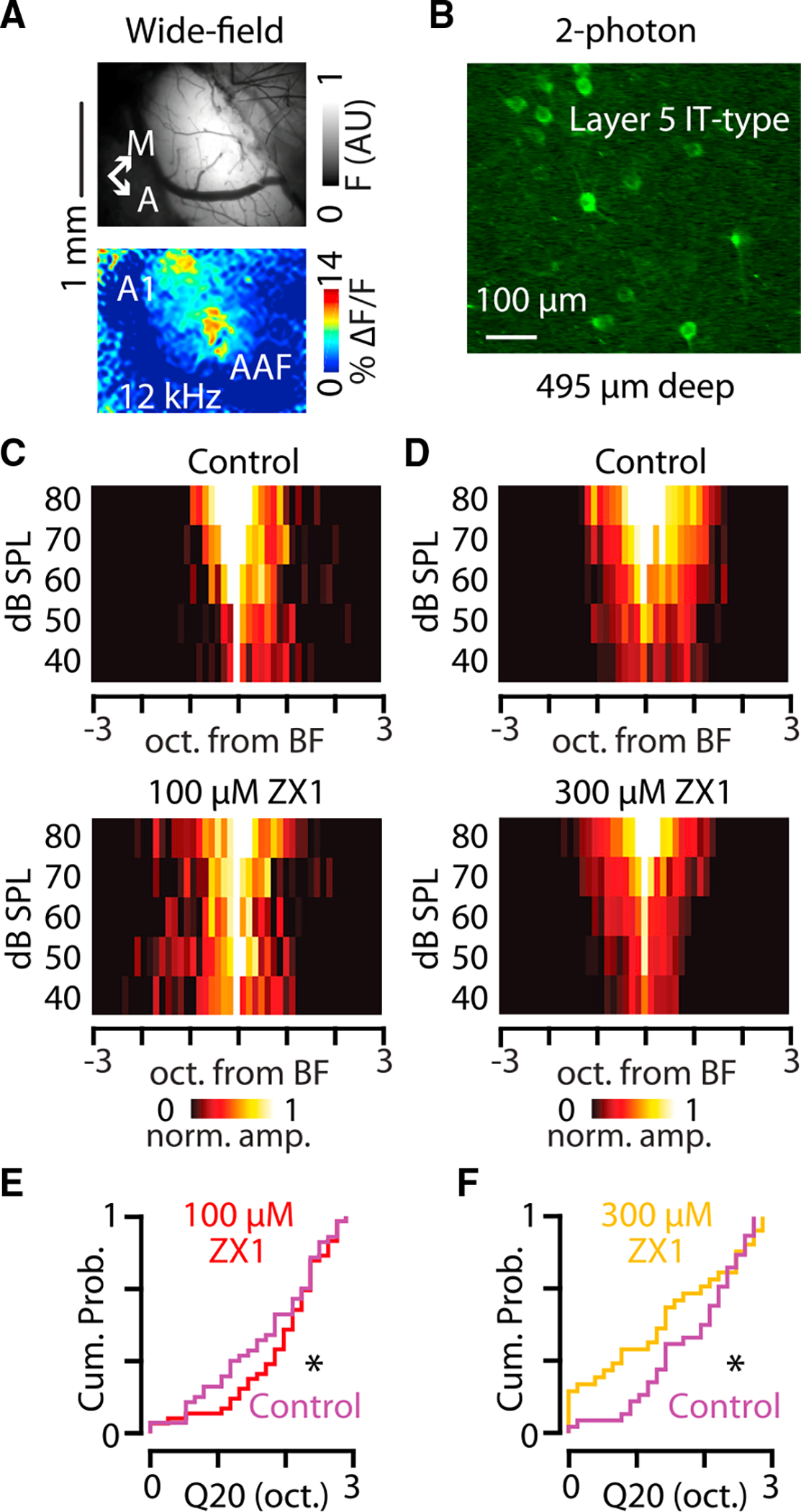
Synaptic zinc can both widen and sharpen sound-frequency tuning bandwidth of layer 5 IT-type neurons in awake mice (A) Top: image of GCaMP7b fluorescence from cortical surface through a craniotomy over the auditory cortex. Bottom: image of the changes in GCaMP7b fluorescence in response to a 50 dB SPL 6 kHz tone presented to the animal. A1, primary auditory cortex; AAF, anterior auditory field. Scale bar: 1 mm. (B) Example 2-photon fluorescent image of somata of layer 5 IT-type neurons in the auditory cortex in an awake mouse. Scale bar: 100 μm. (C) Group data showing the population sound-evoked responses in control (top) and in 100 μM ZX1 (bottom). (D) Group data showing the population sound-evoked responses in control (top) and in 300 μM ZX1 (bottom). (E) Cumulative probability distribution showing the effect of 100 μM ZX1 (red) on the Q20 of layer 5 IT-type neurons (ZX1 vs. control [magenta], p = 0.020, n = 31 neurons from eleven mice; signed-rank test). (F) Cumulative probability distribution showing the effect of 300 μM ZX1 (gold) on the Q20 of layer 5 IT-type neurons (ZX1 vs. control [magenta], p = 0.024, n = 44 neurons from six mice; signed-rank test). Asterisks indicate significant p values. Data are represented as mean ± SEM. See Table S1 for detailed statistics.

Because we observed that synaptic zinc widens the sound-frequency tuning bandwidths of layer 5 PT-type neurons, consistent with the enhancing effects of zinc on AMPAR function at IT-PT synapses, we hypothesized that synaptic zinc could provide concentration-dependent sharpening or widening of sound-frequency tuning bandwidth of IT-type neurons consistent with the bidirectional effects of zinc on AMPAR function at IT-IT synapses. To assess this hypothesis, we used different concentrations of ZX1 to probe bidirectional modulation of sound-frequency tuning bandwidth of IT-type neurons. We injected AAV9-hSyn1-FLEX-jGCaMP7b into the right auditory cortex of PL56-Cre mice (as in Figure 2), or retro-AAV-hSyn1-jGCaMP7b/GCaMP6s into the left auditory cortex of WT mice (as in Figure 3A; STAR Methods). After locating the primary auditory cortex with wide-field calcium imaging (Figure 7A), we used 2-photon calcium imaging to record sound-evoked responses in somata of IT-type neurons in layer 5 (Figure 7B). Consistent with a low amount of zinc chelation resulting in enhancement of AMPAR function at IT-IT synapses, infusions of 100 μM ZX1 resulted in a widening of the bandwidths of IT-type neurons (Figures 7C–7E). Higher amounts of ZX1 (300 μM) had the opposite effect and resulted in a sharpening of sound-frequency tuning bandwidths of IT-type neurons (Figures 7D–7F). These results are consistent with a range of zinc at IT-IT synapses that can be modulated to either widen or sharpen sound-frequency tuning bandwidth of layer 5 IT-type neurons. Together, these results suggest that synaptic zinc signaling supports sound-frequency tuning bandwidths of layer 5 IT- and PT-type neurons in a cell-type-specific manner.

## DISCUSSION

Here, we have described a role for synaptically released zinc to potentiate AMPAR function at specific cortical synapses. These findings complement previously observed inhibitory effects of synaptic zinc on AMPAR function in other brain regions such as the hippocampus and dorsal cochlear nucleus^20,35^ and highlight the role of synaptic zinc on AMPAR function in neocortical circuits. Together, these results suggest that synaptic zinc represents a context-dependent molecular signaling mechanism that can enhance or suppress synaptic strength in a synapse-specific fashion. This is in contrast to zinc’s well-established role to inhibit NMDAR function,^16–18,56–61^ which varies depending on the NMDAR subunit composition^58,84^ but is exclusively inhibitory. The mechanism underlying the modulation of AMPARs by synaptically released zinc is not well understood. AMPAR inhibition by zinc is cyclothiazide insensitive^25,34^ and has no known mechanism, though it has been suggested that this effect may be due to a pore blocking mechanism of zinc ions.^74^ The potentiation of AMPARs by zinc is facilitated by a cyclothiazide-sensitive mechanism,^25,28,46,73,75^ in which zinc is able to potentiate AMPAR potentially by reducing glutamate desensitization of the receptors.^26,85–87^ Because we did not observe effects of ZX1 on the decay kinetics of AMPAR potentiated by endogenous zinc (Figures S5I and S5J), our results suggest that zinc’s effects on decay kinetics may be more difficult to detect in acute brain slices using optogenetic stimulation paradigms, or that other factors governing the clearance and release of synaptic zinc and glutamate might mask this effect.

The reported effects of zinc on AMPAR function are varied, with different studies reaching different conclusions ranging from potentiation, to no effect, to inhibition.^14,17,18,20,24–26,37^ Our findings bring clarity to these apparently contradictory reports about the function of synaptic zinc at different synapses. In this study, we observed that synaptic zinc potentiates AMPAR function at cortical synapses, which is in contrast to previous observations that synaptic zinc inhibits AMPAR function at brainstem and hippocampal synapses^20,35^ and does not affect AMPAR function at cortical and hippocampal synapses.^14,15,17,18^ Our present results offer a simple explanation that unifies these findings because we demonstrate that synaptic zinc can both enhance and suppress AMPAR function at the same synapses, depending on how the effects of zinc are experimentally measured, in particular the concentration of zinc chelator and the pattern of stimulation used. Together, our results show that synaptic zinc can enhance or suppress AMPAR function in a synapse-specific manner, but that both of these effects occur within a potentiating range of zinc levels at these cortical synapses.

The corelease of zinc and glutamate from the same synaptic vesicles is supported by the observation that nearly 100% of synaptic vesicles which contain ZnT3 also contain the vesicular glutamate transporter VGlut1.^1^ These two vesicular membrane transporters demonstrate a synergistic relationship whereby the presence of zinc and ZnT3 can increase the amount of vesicular glutamate,^1,4^ suggesting that ZnT3 containing vesicles may have altered quantal properties compared with those that do not.^1^ Because only about half of synaptic vesicles that express VGlut1 also express ZnT3,^1^ this suggests that although zinc is almost always co-released with glutamate, there are many glutamatergic vesicles that do not corelease zinc. Whether a mix of ZnT3/VGlut1 vs. VGlut1-only vesicles exist within the same synaptic terminal is unknown; however, histological staining of synaptic zinc does not label every vesicle in glutamatergic terminals,^1,2,88–93^ and electrophysiological recordings suggest that ZnT3 containing vesicles may have distinct release machinery than those without ZnT3.^89^ Future studies examining these possibilities will be required, but our results here suggest that different cortical synapses may have differing ratios of ZnT3/VGlut1 vs. VGlut1-only vesicles, which could allow decoupling of glutamate and zinc levels following synaptic release as well as different temporal dynamics for each of these signaling molecules. We observed more pronounced short-term depression with 10 Hz compared with 5 Hz stimulation trains (Figures S1A, S1B, and S6A–S6D); however, the effects of low and high concentrations of ZX1 did not change the relative steady-state level reached during these trains (Figures S6A–S6D). Because the effects of different concentrations of ZX1 are not stimulus-frequency dependent (Figures S6B and S6D), this suggests that at these stimulation frequencies, synaptic zinc does not affect the steady-state level of these trains in an activity-dependent manner. In addition, because synaptic zinc levels can be both increased or decreased by sensory experience^20,94–97^ as well as by patterns of synaptic activity that can cause synaptic plasticity,^36^ our findings suggest that there may be synapse-specific amounts of zinc plasticity at different cortical synapses. Also, as the mechanisms by which zinc is removed from the synaptic cleft are largely unexplored, our results could also be due to synapse-specific differences in the diffusion or clearance of zinc following its release. Future studies examining these possibilities will be required to address these questions.

Our results support and extend previous findings about the effects of synaptically released zinc on the sound-frequency tuning bandwidths of specific classes of cortical neurons. Layer 2/3 IT-type neurons in the primary auditory cortex have sharper tuning bandwidths in the presence of synaptic zinc,^11^ which become wider after zinc chelation with ZX1. As layer 2/3 neurons provide a strong intracortical glutamatergic input to PT-type neurons,^64–70,98–100^ our observations of a sharpening in PT-type tuning bandwidth under similar conditions are consistent with a model by which zinc released from layer 2/3 neurons serves to enhance synaptic strength and couple interlaminar neuronal tuning properties. Our findings suggest that the loss of IT-PT synaptic enhancement after zinc chelation outweighs the wider tuning bandwidth of the presynaptic IT-type neurons, so that the net effect on PT-type tuning is an overall sharpening in sound-frequency tuning bandwidth. As layer 2/3 neurons have low firing rates *in vivo*,^101–104^ our findings suggest that the enhancing effect of zinc on IT-PT synapses is important for the translaminar transfer of sound-evoked information, and that subtle changes in zinc levels could tune these connections for accurate auditory processing. The range of effects of synaptic zinc on IT-IT synapses further supports this interpretation because in experimental regimes that enhance IT-IT synaptic strength, layer 5 IT-type neurons tuning bandwidth is coupled with layer 2/3 IT-type neurons tuning bandwidth, and in regimes where IT-IT synaptic strength is decreased, the populations become decoupled from each other. Although the effects of ZX1 we observed *in vivo* are consistent with the effects of ZX1 we observed in acute brain slices, they are not directly linked to each other, and future studies linking specific synaptic mechanisms to sound-evoked responses will be required to address this issue.

In this study, we have focused on the effects of synaptic zinc between pyramidal cells in the auditory cortex, expanding the known role for zinc signaling in excitatory synaptic transmission. However, the role of zinc signaling on inhibitory circuits of the cortex is not well-characterized. GABAergic interneurons in layer 2/3 of the auditory cortex demonstrate cell-type-specific widening or sharpening of sound-frequency tuning bandwidths by synaptic zinc.^11^ Whereas the organizing principles of inhibitory circuits in superficial cortical layers are relatively well understood,^105–115^ the activation of layer 5 GABAergic interneurons during auditory processing is less well studied. There is evidence that layer 5 somatostatin (SOM) and parvalbumin (PV) interneurons are differentially innervated by layer 2/3 IT-type neurons, and that layer 5 interneurons have distinct inhibitory circuits with IT vs. PT-type neurons.^107,116^ In addition, recent work has demonstrated that SOM interneurons express ZnT3,^37,38,117^ and release synaptic zinc along with GABA.^21^ However, much less is understood about how GABA and synaptic zinc function at inhibitory synapses including the mechanisms of release and the effects on receptor and circuit function. Future work exploring the effects of zinc on inhibitory neurons that shape layer 5 IT- and PT-type neuron sound-evoked properties will be required to address these questions.

### Limitations of the study

In this study, we used AAVs with a synapsin-1 (Syn1) promoter. This promoter is expressed in a broad population of neurons including in layer 2/3 and layer 5 IT-type neurons that also express ZnT3^39,40,117^ and has been used in previous studies of the auditory cortex.^80,82^ Here, we have also used Cre driver lines and long-range axonal projection targets to restrict AAV payload expression to specific and restricted neuronal types in the auditory cortex. However, the unknown extent of overlap between ZnT3 and Syn1 with Sepw1 promoter (used in NP39-Cre mice^43^), Tlx3 promoter (used in PL56-Cre mice^43^), and corticocallosal neurons (used in WT mice) is an important consideration for the interpretation of our results because there may be differences between subpopulations of ZnT3/Syn1-expressing and ZnT3/Syn1-nonexpressing neurons within these cell types. The use of alternative promoters will be required to address this question. This study does not address the roles of zinc signaling on inhibition at the synaptic or systems levels and is focused on the effects of zinc on excitatory synaptic connections in acute brain slices and on the calcium responses of layer 5 excitatory neurons in awake mice. Our current results do not address the potential contribution of inhibitory neuronal activity in shaping the activity of layer 5 IT- and PT-type neurons or the role of synaptic zinc in shaping inhibitory synaptic signaling. Ultimately, this is an important consideration for the interpretation of this study because the sound encoding properties of neurons result from the integration of excitatory drive that is modulated by inhibitory inputs. More detailed studies of the role of zinc on inhibitory signaling will be required to assess these issues.

## STAR★METHODS

Detailed methods are provided in the online version of this paper and include the following:

### RESOURCE AVAILABILITY

#### Lead contact

Further information and requests for resources and reagents should be directed to, and will be fulfilled by, the lead contact, Charles Anderson (charles.anderson@hsc.wvu.edu)

#### Materials availability

This study did not generate any unique reagents.

#### Data and code availability

All data reported in this paper will be shared by the lead contact upon request.

Analyses were performed with commercially available software. No original code was generated or utilized for this paper.

Any additional information required to reanalyze the data reported in this paper is available from the lead contact upon request.

### EXPERIMENTAL MODEL AND STUDY PARTICIPANT DETAILS

#### Mice experiments with electrophysiology and two-photon calcium imaging

Wild-type C57BL/6J mice (Jackson Laboratories), NP39-Cre mice,^43^ PL56-Cre mice,^43^ and ZnT3 KO and WT (Jackson Laboratories) mice were used for these experiments. Animals were maintained in accordance with the animal welfare guidelines and regulations of West Virginia University, the US National Institutes of Health, and the Society for Neuroscience. All procedures were approved by the Institutional Animal Care and Use Committee (IACUC) of West Virginia University, Morgantown, WV. Mice were housed in the West Virginia University Health Sciences Campus Office of Laboratory Animal Research facility, an AAALAC (The Association for Assessment and Accreditation of Laboratory Animal Care International) accredited facility. Same sex littermates were housed together in individual cages with between one and four mice per cage. Mice were maintained on a regular diurnal light cycle (12hr light: 12hr dark) with constant access to food, water, and nesting material for environmental enrichment. Single housed mice were provided with additional enrichment. Animals used for this study were healthy and no previous procedures were performed prior to those described in the methods details.

### METHOD DETAILS

#### Reagents for experiments

##### Basic reagents include

NaCl (Sigma-Aldrich, cat# S9888), Choline chloride (Sigma-Aldrich, cat# C7017), NaHCO_3_ (Sigma-Aldrich, cat# S6014), D-Glucose (Sigma-Aldrich, cat# G8270), sodium ascorbate (Sigma-Aldrich, cat# A7631), sodium pyruvate (Sigma-Aldrich, cat# P8574), KCl (Sigma-Aldrich, cat# P9541), CaCl_2_ (Sigma-Aldrich, cat# 499609), MgCl_2_ (Sigma-Aldrich, cat# 255777), HEPES (Sigma-Aldrich, cat# 54457).

##### Reagents for electrophysiology include

cesium-methanosulfonate (Sigma-Aldrich, cat# C1426), Na_2_-ATP (Sigma-Aldrich, cat# A26209), Tris-GTP (Sigma-Aldrich, cat# G9002), Tris-phosphocreatine (Sigma-Aldrich, cat# P1937), EGTA (Sigma-Aldrich, cat# 324626), CsOH (Sigma-Aldrich, cat# 562505), Na-ascorbate (Sigma-Aldrich, cat# A4034)

#### Brain slice electrophysiology

##### Stereotaxic surgeries

Mice were anesthetized with inhaled isoflurane (induction: 3% in oxygen, maintenance: 1.5% in oxygen) and secured in a stereotaxic frame (Stoelting). Core body temperature was maintained at ~37°C with a heating pad and eyes were protected with ophthalmic ointment. Lidocaine (1%) was injected under the scalp, and an incision was made into the skin at the midline to expose the skull. Using a 27-gauge needle as a scalpel, small craniotomies (~0.4 mm diameter) made over the inferior colliculus at coordinates 1.3 mm posterior and 1.0 mm lateral to lambda, and/or the auditory cortex at coordinates 0.0 mm posterior, 3.7 mm lateral to lambda. Borosilicate glass pipettes (VWR) were pulled to a shallow taper (length >1cm, tip diameter ~30 μm), and were advanced into the region of interest at an angle ~25° off the horizontal plane. Injection pipettes were backfilled with mineral oil (Sigma) and filled with AAV9-flex-Chronos-GFP, retroAAV-hSyn1-GFP-Chronos (Addgene),^48^ or cholera toxin subunit B conjugated to Alexa Fluorophore 555 (CTB-555, 1 mg/sL, Fisher). They were connected to 5 μL glass syringes (Hamilton) via capillary tubing and controlled with syringe pumps (World Precision Instruments). For inferior colliculus injections, pipettes were inserted 1.50 mm deep into the craniotomy and 0.4 μL of CTB-555 was injected at 0.2 μL per minute for 2 min or 0.9 μL of AAV was injected at 0.3 μL per minute for 3 min. For auditory cortex injections, pipettes were inserted 1.30 mm deep into the craniotomy and 0.9 μL of AAV was injected at 0.3 μL per minute for 3 min. After injections, the pipettes were left in place for 2 min prior to removal and then the scalp of the mouse was closed with cyanoacrylate adhesive. Mice received an injection of non-steroidal anti-inflammatory drug meloxicam during the injection procedure, and diet of meloxicam tablets (BioServ) for 72 hr after surgery. Mice were monitored for signs of postoperative stress and pain.

##### Brain slice electrophysiology

Acute brain slice experiments were performed 7 to 12 days post injection with AAV9-flex-Chronos-GFP virus and 14 to 19 days post injection with the retroAAV-hSyn1-GFP-Chronos. Slices were examined during experiments to confirm accurate placement of the injection sites. Brain slices of auditory cortex were cut in chilled carbogenated choline-based solution of the following composition (in mM): 110 Choline chloride, 25 NaHCO_3_, 25 D-Glucose, 11.6 sodium ascorbate, 3.1 sodium pyruvate, 2.5 KCl, 0.5 CaCl_2_, 7 MgCl_2_. Brain slice electrophysiology experiments were carried out using carbogenated artificial cerebrospinal fluid (ACSF) with the following composition (in mM): 130 NaCl, 3 KCl, 2.4 CaCl_2_, 1.3 MgCl_2_, 20 NaHCO_3_, 3 HEPES, 10 D-glucose, saturated with 95% O_2_/5% CO_2_ (v/v), pH 7.25–7.35, ~300 mOsm. All solutions were continuously bubbled with carbogen. For experiments described in Figure S2, experiments were performed using ACSF containing 50 μM MgCl_2_. Contaminating zinc was removed from the ACSF for all experiments by stirring the ACSF with Chelex 100 resin (Biorad) for 1 h. High purity CaCl_2_, and MgCl_2_ salts (99.995% purity; Sigma Aldrich) were added to the ACSF after the Chelex resin was filtered using Nalgene rapid flow filters lined with polyethersulfone (0.2 μM pore size). All plastic and glassware were washed with 5% high purity nitric acid. Experiments with ZnT3 KO mice were performed blind to their genotype. Mice were first anesthetized with isoflurane and then immediately decapitated. Brains were rapidly removed and coronal slices (300 μm) of the cortex were prepared in chilled choline chloride cutting solution using a vibratome (VT1200 S; Leica). Slices were then transferred into a holding chamber of carbogenated ACSF and incubated for ~30 min at 35°C and then incubated at room temperature for ~30 min before electrophysiological experiments were performed. For electrophysiological experiments, slices were transferred into the recording chamber and perfused with carbogenated ACSF at a rate of 1–2 mL/min. Recordings were performed at 30°C–32°C using an in-line heating system (Warner Instruments). Corticocollicular neurons were identified by their labeling with the retrograde labeler Cholera Toxin Subunit B conjugated to Alexa 555 (Fisher). Images were prepared using ImageJ (Fiji). Electrophysiological recordings were made using an amplifier (MultiClamp-700B, Axon Instruments), a digital to analog converter (USB-6229, National Instruments), and ephus.^118^ Voltage-clamp recordings were conducted using borosilicate pipettes (Warner Instruments) pulled to tip resistances of 3–5 MΩ (Sutter Instruments) filled with a cesium-based internal solution with the following composition (in mM): 128 cesium-methanosulfonate, 10 HEPES, 4 MgCl_2_, 4 Na_2_-ATP, 0.3 Tris-GTP, 10 Tris-phosphocreatine, 0.5 cesium-EGTA, 3 Na-ascorbate, 1 QX314. (pH = 7.23, 303 mOsm) at −70mV holding. ZX1 (50, 100, or 300 μM), AP5 (50 μM), DNQX (20 μM), BCS (50 μM), and cyclothiazide (20 μM) were bath applied. We pharmacologically confirmed that EPSCs were mediated by AMPARs with the addition of the AMPAR antagonist DNQX at the end of every recording. For NMDAR experiments, a modified low-magnesium ACSF (containing 50 μM MgCl_2_) was used to relieve the magnesium block of NMDARs at −70 mV holding potential. In these conditions EPSCs had a long decay tau consistent with mixed activation of both AMPAR and NMDAR. The addition of the AMPAR specific antagonist DNQX reduced the peak amplitude and delayed the time of the response peak, but did not affect the longer decay tau, suggesting that this component of the EPSC was mediated by NMDARs, which are slower to activate and slower to deactivate than AMPARs.^119^ We pharmacologically confirmed that DNQX insensitive component of the EPSC was mediated by NMDARs with addition of the NMDAR specific antagonist AP5 at the end of every recording (Figures S2B–S2D).

#### Experimental design

Experiments were replicated within and between mice. ZnT3 KO experiments were performed blind to genotype. The number of replicates per experiment and their definitions are listed in the figure legends. Experiments were excluded if the series resistance was above 30 MΩ or changed more than 15% during the recording session. For experiments involving multiple concentrations of ZX1, some experiments were performed sequentially, while others were performed with only a single concentration to account for time-related changes in recordings.

#### Analysis of electrophysiological data

Data were sampled at 10 kHz and lowpass filtered at 2–4 kHz. AMPAR EPESCs in neurons were evoked by stimulation of the opsin-expressing neurons with 1 msec long pulses of 470 nm blue light delivered through the microscope objective using a CoolLED pE-300^white^ (CoolLED). For all experiments, series resistance was monitored during the experiment by giving brief −5 mV voltage steps at regular intervals during the recording session. Analysis was performed using MATLAB (MathWorks). For all experiments, traces shown are the average of recordings in each condition (three to seven trials). Amplitude data were quantified from these averaged responses. EPSC amplitudes were quantified as the peak-to-trough amplitude, measured as the difference between the current amplitude at the start of each light pulse and the peak of EPSC following each light pulse. AMPAR EPSC decay time constants (decay tau) were calculated by fitting a single exponential to the decay phase of the response. For graphs in figures: Figures 1G, 2C, 3D, 4B, 5EK, S2C, S3B, S4BG, S7B and S7C, each data point represents the amplitude of the EPSC normalized to the amplitude of the control EPSC (EPSC_1_/Control_1_). For graphs in figures: Figures 1J, 2E, 3G, 4D and S3D, each data point represents the amplitude of each EPSC during the train normalized to the amplitude of the first control EPSC (EPSC_n_/Control_1_). For graphs in figures: Figures 1K, 2F, 3H, 4E, 5GM, S3E and S5BF, each data point represents the amplitude of the post-treatment EPSC normalized to the amplitude of the corresponding control EPSC (EPSC_n_/Control_n_). For graphs in figures: Figures 5HN, S4EJ and S5CG, each data point represents the average amplitude of the EPSCs along a stimulus train, normalized to the average amplitude of EPSCs along a stimulus train in the control condition (EPSC_avg_/Control_avg_). For graphs in figures: Figures S1A, S1B and S5A–S5D, each point represents the amplitude of EPSC for each stimulus normalized to the amplitude of the first EPSC amplitude for that train (EPSC_n_/EPSC_1_). Data from neurons in Figure 4BG are also used in Figures 5KM and S4F.

#### Two-photon imaging

##### Stereotaxic surgeries

Mice were anesthetized with inhaled isoflurane (induction: 3% in oxygen, maintenance: 1.5% in oxygen) and secured in a stereotaxic frame (Stoelting). Core body temperature was maintained at ~37°C with a heating pad and eyes were protected with ophthalmic ointment. Lidocaine (1%) was injected under the scalp, and an incision was made into the skin at the midline to expose the skull. Using a 27-gauge needle as a scalpel, small craniotomies (~0.4 mm diameter) made over the inferior colliculus at coordinates 1.3 mm posterior and 1.0 mm lateral to lambda, and/or the auditory cortex at coordinates 0.0 mm posterior, 3.7 mm lateral to lambda. Borosilicate glass pipettes (VWR) were pulled to a shallow taper (length >1cm, tip diameter ~30 μm), and were advanced into the region of interest at an angle ~25° off the horizontal plane. Injection pipettes were backfilled with mineral oil (Sigma) and filled with AAV9-hSyn1-FLEX-jGCaMP7b, retro-AAV-hSyn1-jGCaMP7b,^120^ or retro-AAV-hSyn1-GCaMP6s^77^ (titer 5e^12^ – 5e^13^ genome copies/mL, Addgene). They were connected to 5 μL glass syringes (Hamilton) via capillary tubing and controlled with syringe pumps (World Precision Instruments). For inferior colliculus injections, pipettes were inserted 1.50 mm deep into the craniotomy and 0.4 μL of AAV was injected at 0.2 μL per minute for 2 min or 0.9 μL of AAV was injected at 0.3 μL per minute for 3 min. For auditory cortex injections, pipettes were inserted 1.30 mm deep into the craniotomy and 0.9 μL of AAV was injected at 0.3 μL per minute for 3 min. After injections, the pipettes were left in place for 2 min prior to removal and then the scalp of the mouse was closed with cyanoacrylate adhesive. Mice received an injection of non-steroidal anti-inflammatory drug meloxicam during the injection procedure, and diet of meloxicam tablets (BioServ) for 72 h after surgery. Mice were monitored for signs of postoperative stress and pain.

#### Craniotomy

11–24 days after AAV injections, mice were prepared for *in vivo* calcium imaging. Mice were anesthetized with inhaled isoflurane (induction: 3% in oxygen, maintenance: 1.5% in oxygen) and positioned into a custom head holder. Core body temperature was maintained at ~37°C with a heating pad and eyes were protected with ophthalmic ointment. Lidocaine (1%) was injected under the scalp and an incision (~1.5 cm long) was made into the skin over the right temporal cortex. The head of the mouse was rotated ~45° in the coronal plane to align the pial surface of the right temporal cortex with the imaging plane of the upright microscope optics. The skull of the mouse was secured to the head holder using dental acrylic (Lang) and cyanoacrylate adhesive. A tube (the barrel of a 25 mL syringe or an SM1 tube from Thorlabs) was placed around the animal’s body to reduce movement, and the mouse received an injection of chlorprothixene (1.5 mg/kg intraperitoneal). A dental acrylic reservoir was created to hold warm artificial cerebrospinal fluid (ACSF) over the exposed skull. In preparing the ACSF, we removed contaminating zinc by incubating with Chelex 100 resin (Biorad) for 1 h, removed the Chelex by vacuum filtration, and then added high purity calcium and magnesium salts (99.995% purity; Sigma-Aldrich). The solution contained in millimolar: 130 NaCl, 3 KCl, 1.2 CaCl_2_$2H_2_O, 1.3 MgCl_2_$6H_2_O, 20 NaHCO_3_, 3 HEPES, and 10 D-glucose, pH = 7.25–7.35, ~300 mOsm. We performed a craniotomy (~1 mm^2^) of the skull over the temporal cortex and mice were then positioned under the microscope objective in a sound and light attenuation chamber containing the microscope and a calibrated speaker (ES1, Tucker-Davis). Using a micromanipulator (Siskiyou), we inserted a glass micropipette backfilled with mineral oil and connected to a 5 μL glass syringe into the cortex at the edge of this craniotomy (Figure 1C left). The pipette contained ACSF including 100 μM of ZX1 and 50 μM Alexa 594. Once the pipette was inserted into the cortex, we then removed the isoflurane from the oxygen flowing to the animal and began imaging sound-evoked responses at least 20 min later.^11,12,21,29,78^

#### Calcium imaging

We imaged the change in green GCaMP emission with epifluorescence optics (eGFP filter set, U-N41017, Olympus) and a 4× objective (Olympus) using a cooled CCD camera (Rolera, Q-Imaging). Images were acquired at a resolution of 200 × 150 pixels (using 8X spatial binning, each pixel covered an area of = 222.2 μm^2^ of the image) at a frame rate of 20 Hz. To locate A1 we normalized the sound-evoked change in fluorescence after sound presentation (ΔF) to the baseline fluorescence (F), where F is the average fluorescence of 1 sec preceding the sound onset (for each pixel in the movie). We applied a two-dimensional, low-pass Butterworth filter to each frame of the ΔF/F movie, and then created an image consisting of a temporal average of 10 consecutive frames (0.5 sec) beginning at the end of the sound stimulus. This image indicated two sound-responsive regions corresponding to the low-frequency tonotopic areas of A1 and the AAF.

After locating A1, we then performed 2-photon imaging of GCaMP-expressing neuronal somata in layer 5 of A1. Mode-locked infrared laser light (940 nm, 100–200 mW intensity at the back focal plane of the objective, Insight ×3, Newport) was delivered through a galvanometer-based scanning 2-photon microscope (Scientifica) controlled with scanimage 3.8,^121^ using a 16X, 0.8 NA objective (Nikon) with motorized stage and focus controls. We imaged green and red fluorescence simultaneously with 2 photomultiplier tubes behind red and green emission filters (FF01-593/40, FF03-525/50, Semrock) using a dichroic splitter (Di02-R561, Semrock) at a frame rate of 5 Hz over an area of 244 μm × 227 μm and at a resolution of 256 × 240 pixels. We imaged neurons in layer 5 at depths of 450–600 μm from pia. After identifying A1 neurons responding to sounds, we presented stimulus blocks containing different intensities and frequencies of sounds (40, 50, 60, 70, 80 dB SPL; 5–40 kHz in 1/8 octave steps, 500 msec duration, 20 msec ramps) while monitoring the changes in GCaMP fluorescence. Each stimulus block contained 125 different combinations of frequencies and intensities and each unique sound was presented pseudorandomly with 3 sec interstimulus intervals using wavesurfer 1.0.6 (Janelia Farms) to drive the speaker as above. Each animal received 5–7 presentations of each stimulus block. After obtaining movies of responses to different sound stimuli, we infused ZX1 solution into the cortex at a rate of 120 nL/min and remeasured the responses of the same neurons to the same sounds. Mice were euthanized at the end of the recording session.

#### Experimental design

Experiments were replicated within and between mice. The number of replicates per experiment and their definitions are listed in the figure legends. Acoustic stimuli were calibrated with ¼ inch microphone (Brüel & Kjær) placed at the location of the animal’s ear within the chamber. Sounds were delivered from a free-field speaker ~10 cm from the left ear of the animal (MF1 speaker, SA1 driver, Tucker-Davis Technologies), controlled by a digital to analog converter with an output rate of 250 kHz (USB-6343, National Instruments). We used ephus^118^ to generate the sound waveforms and synchronize the sound delivery and image acquisition hardware. To locate A1, we presented 50 or 60 dB SPL, 6 kHz tones to the animal while illuminating the cortex with a blue LED (nominal wavelength of 490 nm, M490L2, Thorlabs).

#### Analysis of 2-photon imaging data

To quantify the neuronal responses to sounds, we identified neurons that were present in the field of view before and after ZX1 infusion and targeted only these cells for analysis. Using FluoroSNNAP software,^11,80,122^ we selected ROIs within the soma of each neuron in each block of stimulation. The pixels in each ROI from each frame were averaged and converted into ΔF/F as above. We then averaged the fluorescent response for 5–7 presentations of the same sound intensity and frequency for each neuron and converted the average response into *Z* score by subtracting the mean of the entire trace from each time point and dividing that value by the standard deviation of the entire trace. Sound-evoked responses were measured for 1 sec after the sound onset and were defined as responses if the sound-evoked changes in fluorescence were larger than 0.6 *Z* score for at least two consecutive frames. Responses were quantified as the peak of the increased fluorescence during this 1 sec period. Best frequency (BF) was defined as the sound frequency resulting in the largest response independent of sound intensity.^79^ Threshold was defined as the lowest sound intensity that resulted in a sound-evoked response. Receptive field bandwidth (Q20) was defined as log_2_ of the ratio of the highest sound frequency and the lowest sound frequency that elicited a response from the neuron 20 dB above threshold. We calculated the d-prime for each neuron^81,123^ and focused the top 20% of sound-responsive neurons for analysis of sound-frequency tuning bandwidths.

### QUANTIFICATION AND STATISTICAL ANALYSIS

For every comparison in the manuscript, we describe the statistical test used, the exact value of n, what n represents, the measure of central tendency (e.g., median or mean), and the definition of the error bars. Statistical details are included in the figure legend corresponding to where the data are presented, as well as in the results section. A statistical comparison was defined to be significant if the p value was less than 0.05 (for single comparisons) or by use of the Holm-Bonferroni correction (for multiple comparisons). All significant statistical values are marked by an asterisk (*) in figures, and p values are listed in the figure legends. Error bars represent ± standard error of the mean (SEM). Exclusion criteria are listed in the corresponding methods section when appropriate. Number of cells and mice are reported for each experiment.

For statistical comparisons, Student paired t test, Student unpaired t test, and 2-way repeated measures ANOVA were used if the group data passed Lilliefor’s test for normality. Welch’s correction for unequal variance was applied when appropriate. If the group data were not normally distributed, then the Wilcoxon signed-rank test was used. Significance levels were determined based on Holm-Bonferroni post hoc correction for multiple comparisons. Statistical analyses were performed with Prism (GraphPad) or MATLAB (Mathworks). Experiments were designed in a paired fashion (e.g., treatment compared to control for each neuron) and normalized to control values to reduce the influence of cell-to-cell variability and increase the statistical power of the study by allowing the use of paired statistical tests when possible. The number of replicates per experimental group was not predetermined, but are consistent with those reported from similar studies in the field.^15–18,20^

## Supplementary Material

1

2

## Figures and Tables

**KEY RESOURCES TABLE:** 

REAGENT or RESOURCE	SOURCE	IDENTIFIER

Bacterial and virus strains		

AAV9-flex-Chronos-GFP	Addgene (Klapoetke et al., 2014)^48^	Cat#: 84482
retroAAV-hSyn1-GFP-Chronos	Addgene (Klapoetke et al., 2014)^48^	Cat#: 59170
AAV9-hSyn1-FLEX-jGCaMP7b	Addgene (Dana et al., 2019)^120^	Cat#: 104493
retro-AAV-hSyn1-jGCaMP7b	Addgene (Dana et al., 2019)^120^	Cat#: 104489
retro-AAV-hSyn1-GCaMP6s	Addgene (Chen et al., 2013)^77^	Cat#: 51084

Chemicals, peptides, and recombinant proteins		

Cholera toxin subunit B conjugated to Alexa fluorophore 555	Thermo Fisher Scientific	Cat#: C34776
ZX1	Strem Chemicals	Cat#: 07-0350
DNQX	Sigma-Aldrich	Cat#: 505026
QX314	hellobio	Cat#: HB1030
cyclothiazide	Sigma-Aldrich	Cat#: c9847
Bathocuproine sulfonate	Fisher Scientific	Cat#: AC164060010

Experimental models: Organisms/strains		

Mouse: C57BL/6J	The Jackson Laboratory	RRID:IMSR_JAX:000664
Mouse: B6; 129-*Slc30a3*^*tm1Rpa*^/J (ZnT3 KO)	The Jackson Laboratory	RRID:IMSR_JAX:005064
Mouse: Sepw1(NP39)-cre	The Jackson Laboratory	MGI:5519915
Mouse: Tg(Tlx3-cre)PL56Gsat	The Jackson Laboratory	RRID:MGI:5312969

Software and algorithms		

ImageJ	FIJI	RRID:SCR_003070
MATLAB	Mathworks	RRID:SCR_001622
ephus	(Suter et al., 2010)^118^	N/A
GraphPad Prism 9	GraphPad	RRID:SCR_000306
scanimage	Vidrio Technologies	RRID:SCR_014307
wavesurfer	Janelia Farms	RRID:SCR_021529
FluoroSNNAP	(Patel, et al. 2015)^122^	N/A

## Highlights

- Synaptic zinc potentiates AMPAR function in the auditory cortex
- The effects of synaptic zinc on AMPAR function are cell-type and synapse specific
- Synaptic zinc can enhance or suppress AMPAR function
- Synaptic zinc can sharpen or broaden sound-frequency tuning bandwidths of layer 5 neurons

## INCLUSION AND DIVERSITY

We worked to ensure sex balance in the selection of non-human subjects. One or more of the authors of this paper self-identifies as living with a disability. While citing references scientifically relevant for this work, we also actively worked to promote gender balance in our reference list. We support inclusive, diverse, and equitable conduct of research.
