# Supplementary material for: Synaptic zinc potentiates AMPA receptor function in mouse auditory cortex
Source: Cell Rep. Author manuscript; Available in PMC 2023 Sep 22. (PMC10514716; doi:10.1016/j.celrep.2023.112932)
Supplement: 1 [file NIHMS1928263-supplement-1.pdf]

Cell Reports, Volume *42*

Supplemental information

Synaptic zinc potentiates AMPA

receptor function in mouse auditory cortex

Philip T.R. Bender, Mason McCollum, Helen Boyd-Pratt, Benjamin Z. Mendelson, and Charles T. Anderson

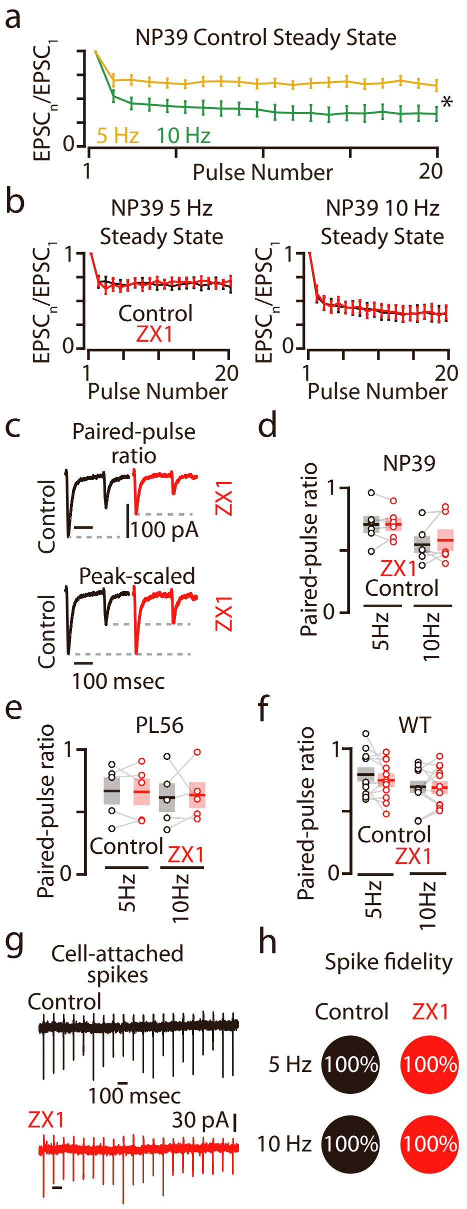
a
NP39 Control Steady State
EPSC_n/EPSC_1
5 Hz
10 Hz
Pulse Number
b
NP39 5 Hz Steady State
NP39 10 Hz Steady State
Control
ZX1
Pulse Number
c
Paired-pulse ratio
Control
ZX1
100 pA
Peak-scaled
100 msec
d
NP39
Paired-pulse ratio
5Hz
10Hz
e
PL56
f
WT
g
Cell-attached spikes
100 msec
30 pA
h
Spike fidelity
5 Hz
10 Hz
100%

Figure S1

Figure S1: Endogenous zinc has post-synaptic effects on AMPAR EPSCs. Related to Figures 1, 2, and 4.

a) Average NP39 control AMPAR EPSC steady state along a stimulus train of twenty pulses. Data shown as $EPSC_n/EPSC_1$, normalized EPSC amplitudes compared at 5 Hz stimulation (yellow) and 10 Hz stimulation (green). (p=0.0029, n=6 cells from six mice. Two-way repeated measures ANOVA.)

b) Average NP39 control AMPAR EPSC steady state along a stimulus train of twenty pulses. Data shown as $EPSC_n/EPSC_1$ Normalized EPSC amplitudes compared at 5 Hz (left) stimulation and 10 Hz stimulation (right) in control (black) and after 100 µM ZX1 (red). (5 Hz: p=0.6882, n=6 cells from six mice. Two-way repeated measures ANOVA. 10 Hz: p=0.7677, n=6 cells from six mice. Two-way repeated measures ANOVA.)

c) (Top) Example EPSCs pairs from the same neuron in control (black) and in ZX1 (red). (Bottom) The same traces peaked scaled. Color scheme as in **b**.

d) Average effect of ZX1 on paired pulse ratios of NP39 at 5 Hz (left) and 10 Hz (right). (5 Hz: p=0.4991, n=6 cells from 4 mice. 10 Hz: p=0.6981, n=6 cells from four mice. Paired t-test.). Color scheme as in **b**.

e) Average effect of ZX1 on paired pulse ratios of PL56 at 5 Hz (left) and 10 Hz (right). (5 Hz: p=0.8770, n=5 cells from 4 mice; 10 Hz: p=0.8289, n=5 cells from four mice. Paired t-test). Color scheme as in **b**.

f) Average effect of ZX1 on paired pulse ratios of WT at 5 Hz (left) and 10 Hz (right). (5 Hz: p=0.6031, n=12 cells from 10 mice. 10 Hz: p=0.9223, n=12 cells from ten mice. Paired t-test.). Color scheme as in **b**.

g) Example traces showing light-evoked action potentials of a layer 2/3 chronos-expressing neuron recorded in cell-attached mode in control (black) and in ZX1 (red).

h) Average light-evoked spike fidelity of layer 2/3 neurons to twenty light pulses at 5 Hz (top) and 10 Hz (bottom) in control (black) and in ZX1 (red).

Asterisks indicate significant p values. Data are represented as mean ± SEM. See Table S1 for detailed statistics.

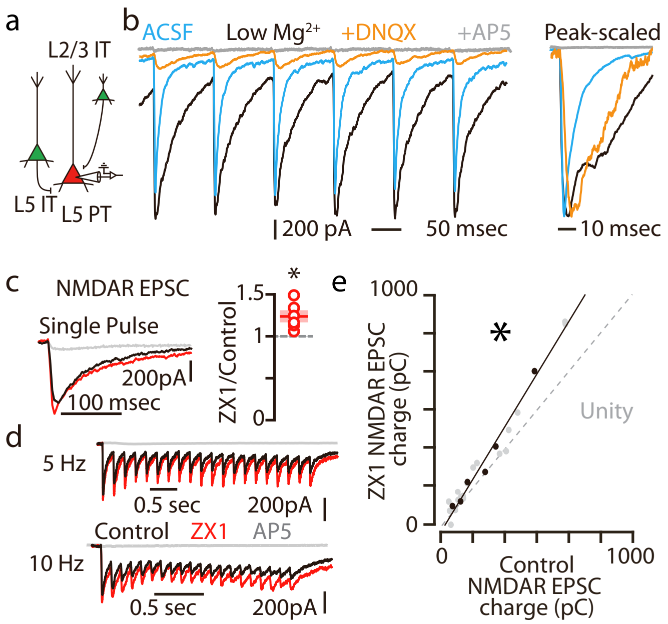

a
L2/3 IT
L5 IT
L5 PT
b
ACSF
Low Mg2+
+DNQX
+AP5
Peak-scaled
200 pA
50 msec
10 msec
c
NMDAR EPSC
Single Pulse
200pA
100 msec
ZX1/Control
1.5
1
0
*
d
5 Hz
0.5 sec
200pA
Control
ZX1
AP5
10 Hz
0.5 sec
200pA
e
1000
0
ZX1 NMDAR EPSC charge (pC)
*
Unity
0
1000
Control NMDAR EPSC charge (pC)

Figure S2

Figure S2. Synaptic zinc inhibits NMDA receptor function at IT-PT synapses in the auditory cortex. Related to Figures 4 and S3.

a) (Left) Cartoon illustrating presynaptic IT-type neurons expressing chronos (green) and a post synaptic layer 5 neuron expressing CTB-555 (red).

b) (Left) Example traces showing averaged EPSCs in response to pulses of blue light. Blue: AMPAR EPSCs in regular ASCF, Black: EPSCs from activation of both AMPAR and NMDAR in low magnesium ACSF. Gold: NMDAR EPSCs in low magnesium ACSF and the AMPAR antagonist DNQX. Gray: EPSC in low magnesium ACSF, DNQX, and the NMDAR antagonist AP5. (Right) Example traces showing peak-scaled EPSCs, highlighting that EPSCs isolated with DNQX in low-magnesium conditions (gold) showed a slower rise time and longer tau than isolated AMPAR EPSCs (blue) consistent with these EPSCs being mediated by NMDARs.

c) (Left) Example traces showing averaged NMDAR EPSCs in response to a single pulse of blue light. Black: control EPSC. Red: EPSC after the addition of ZX1. Gray: EPSC after the addition of AP5. (Right) Average effect of ZX1 on the amplitude of NMDAR EPSCs in response to a single pulse of light ($p=0.0144$, $n=6$ cells from five mice, paired t-test).

d) Example averaged EPSCs from trains of light pulses at 5 Hz (top) and 10 Hz (bottom). Color scheme same as in **c**.

e) Average effect of ZX1 on the charge of averaged NMDAR EPSCs. Gray: charge of individual EPSCs in response to three light stimulations (single pulse, 5 Hz, and 10 Hz). Black: Average charge of all EPSCs from each neuron ($p=0.0080$, $n=6$ cells from five mice, grouped t-test). At all stimulation frequencies, we observed a significant increase in the first peak amplitude and charge of NMDAR EPSCs after zinc chelation with ZX1, suggesting that the synaptic zinc concentration is sufficient to inhibit NMDAR EPSCs at the same layer 2/3 IT-PT and layer 5 IT-PT synapses, where it is also able to potentiate AMPAR EPSCs (Figure 4).

Asterisks indicate significant p values. Data are represented as mean ± SEM. See Table S1 for detailed statistics.

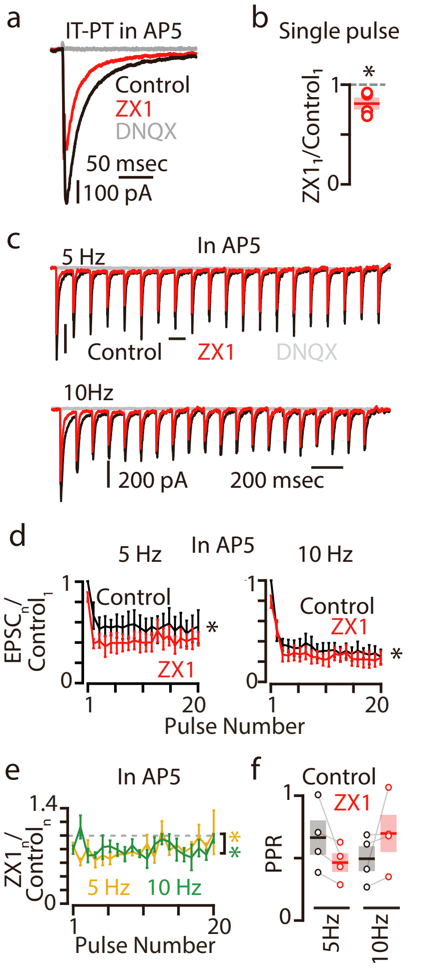

a
IT-PT in AP5
Control
ZX1
DNQX
50 msec
100 pA
b
Single pulse
ZX1₁/Control₁
1
0
*
c
5 Hz
In AP5
Control
ZX1
DNQX
10Hz
200 pA
200 msec
d
5 Hz
In AP5
10 Hz
EPSCn / Control1
Control
ZX1
Pulse Number
e
In AP5
1.4
ZX1n / Controln
5 Hz
10 Hz
Pulse Number
f
Control
ZX1
PPR
5Hz
10Hz

Figure S3

Figure S3. AMPA receptor function enhancement by synaptic zinc does not depend on NMDA receptor signaling. Related to Figures 4 and S2.

a) Example traces showing average AMPAR EPSCs in response to a single pulse of blue light in the presence of the NMDAR antagonist AP5. Black: control EPSC. Red: EPSC after the addition of ZX1. Gray: EPSC after the addition of the AMPAR antagonist DNQX.

b) Average effect of ZX1 on the average amplitude of AMPAR EPSCs in response to a single pulse of light in the presence of AP5 (p=0.0486, n=4 cells from four mice, paired t-test). Color scheme as in **a**.

c) Example average EPSCs from trains of light pulses at 5 Hz (top) and 10 Hz (bottom). Color scheme as in **a**.

d) Average effect of ZX1 on the average amplitude of AMPAR EPSCs normalized to the first EPSC amplitude in response to trains of light pulses at 5 Hz (left) and 10 Hz (right) (5 Hz: p=0.0276, n=4 cells from four mice; 10 Hz: p=0.0183, n=4 cells from four mice; 2-way repeated measures ANOVAs). Color scheme as in **a**.

e) Average effect of ZX1 on the average amplitude of AMPAR EPSCs normalized to the amplitude of each corresponding control EPSC in response to trains of light pulses at 5 Hz (yellow) and 10 Hz (green) (5 Hz: p=0.0135, n=4 cells from four mice; 10 Hz: p=0.0319, n=4 cells from four mice; 2-way repeated measures ANOVAs).

f) Average effect of ZX1 on paired pulse ratios at 5 Hz (left) and 10 Hz (right). Color scheme as in **a**.

Asterisks indicate significant p values. Data are represented as mean ± SEM. See Table S1 for detailed statistics.

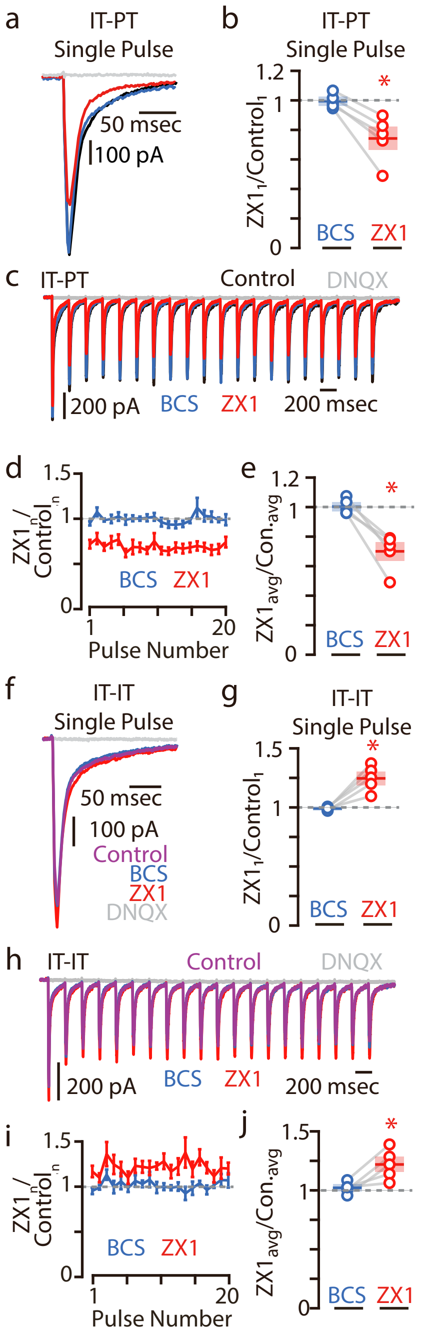
a
IT-PT
Single Pulse
50 msec
100 pA
b
IT-PT
Single Pulse
1.2
1
0
ZX1$_1$/Control$_1$
*
BCS
ZX1
c
IT-PT
Control
DNQX
200 pA
BCS
ZX1
200 msec
d
1.5
1
0
ZX1$_n$/Control$_n$
BCS
ZX1
1
20
Pulse Number
e
1.2
1
0
ZX1$_{avg}$/Con.$_{avg}$
*
BCS
ZX1
f
IT-IT
Single Pulse
50 msec
100 pA
Control
BCS
ZX1
DNQX
g
IT-IT
Single Pulse
1.5
1
0
ZX1$_1$/Control$_1$
*
BCS
ZX1
h
IT-IT
Control
DNQX
200 pA
BCS
ZX1
200 msec
i
1.5
1
0
ZX1$_n$/Control$_n$
BCS
ZX1
1
20
Pulse Number
j
1.5
1
0
ZX1$_{avg}$/Con.$_{avg}$
*
BCS
ZX1

Figure S4

Figure S4. Copper does not modulate AMPAR EPSC function or occlude the effects of ZX1 at IT-IT or IT-PT synapses. Related to Figures 3 and 4.

a) Example traces showing average AMPAR EPSCs in response to a single pulse of blue light at IT-PT synapses. Black: control EPSC. Blue: EPSC in the presence of BCS. Red: EPSC in the presence of BCS and ZX1. Gray: EPSC after the addition of DNQX.

b) Average effect of BCS and subsequent addition of ZX1 on the average amplitude of IT-PT AMPAR EPSCs in response to a single pulse of light (BCS: p=0.8146, n=5 cells from five mice; ZX1: p=0.0061, n=5 cells from five mice, paired t-test). Color scheme as in **a**.

c) Example average EPSCs from trains of light pulses at IT-PT synapses. Color scheme as in **a**.

d) Average effect of ZX1 on the average amplitude of IT-PT AMPAR EPSCs normalized to the amplitude of each corresponding control EPSC in response to trains of light pulses after the addition of BCS and the subsequent addition of ZX1. Color scheme as in **a**.

e) Average effect of ZX1 on the amplitude of average IT-PT AMPAR EPSCs in response to a stimulus blue light normalized to the corresponding control EPSC after the addition of BCS and the subsequent addition of ZX1 (BCS: p=0.9248, n=5 cells from five mice; ZX1: p=0.9.4647e-4, n=5 cells from five mice. Paired t-test.) Color scheme as in **a**.

f) Example traces showing average AMPAR EPSCs in response to a single pulse of blue light in the presence of BCS and ZX1 recorded from IT-IT synapses. Magenta: control EPSC. Blue: EPSC in the presence of BCS. Red: EPSC in the presence of BCS and ZX1. Gray: EPSC after the addition of DNQX.

g) Average effect of BCS and subsequent addition of ZX1 on the average amplitude of IT-IT AMPAR EPSCs in response to a single pulse of light (BCS: p=0.1640, n=5 cells from five mice; ZX1: p=0.0109, n=5 cells from five mice. Paired t-test). Color scheme as in **f**.

h) Example average EPSCs from trains of light pulses at IT-IT synapses. Color scheme as in **f**.

i) Average effect of ZX1 on the average amplitude of IT-IT AMPAR EPSCs normalized to the amplitude of each corresponding control EPSC in response to trains of light pulses after the addition of BCS and the subsequent addition of ZX1. Color scheme as in **f**.

j) Average effect of ZX1 on the amplitude of average IT-IT AMPAR EPSCs in response to a stimulus blue light normalized to the corresponding control EPSC after the addition of BCS and the subsequent addition of ZX1 (BCS: p=0.4518, n=5 cells from five mice; ZX1: p=0.0061, n=5 cells from five mice. Paired t-test.) Color scheme as in **f**.

Asterisks indicate significant p values. Data are represented as mean ± SEM. See Table S1 for detailed statistics.

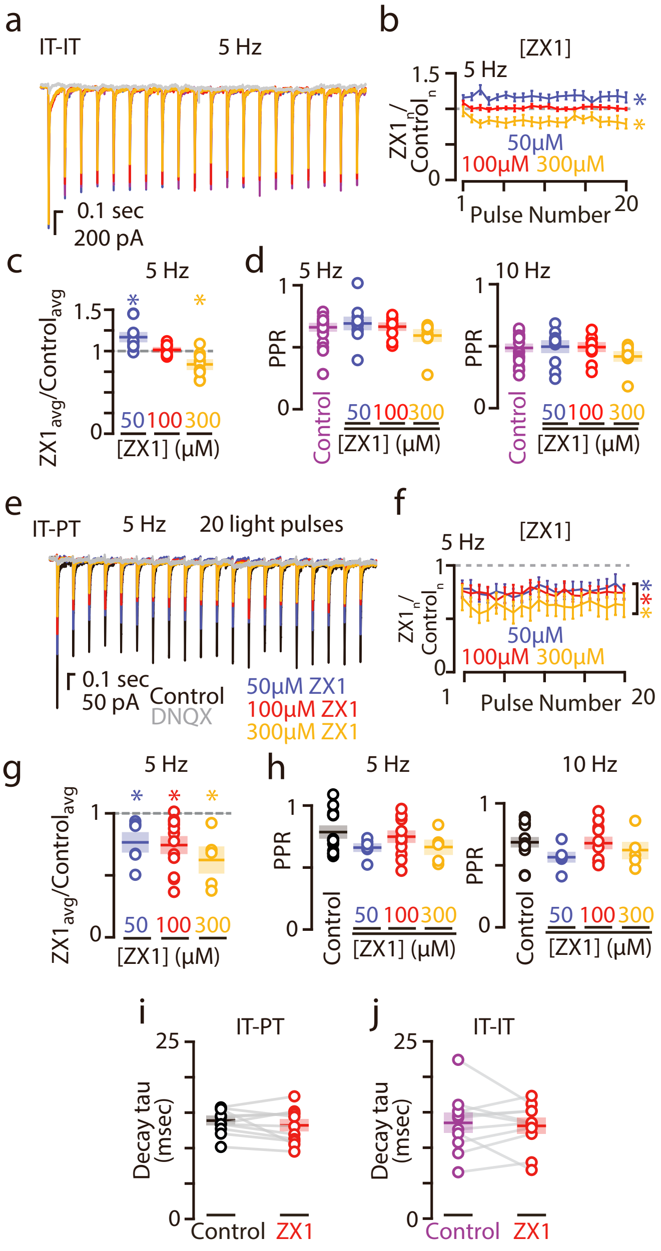

a
IT-IT
5 Hz
0.1 sec
200 pA
b
[ZX1]
5 Hz
1.5
0
ZX1n/Controln
50μM
100μM 300μM
Pulse Number
1
20
c
5 Hz
1.5
1
0
ZX1avg/Controlavg
50 100 300
[ZX1] (μM)
d
5 Hz
10 Hz
1
0
PPR
Control
50 100 300
[ZX1] (μM)
e
IT-PT
5 Hz
20 light pulses
0.1 sec
50 pA
Control
DNQX
50μM ZX1
100μM ZX1
300μM ZX1
f
5 Hz
[ZX1]
1
0
ZX1n/Controln
50μM
100μM 300μM
Pulse Number
1
20
g
5 Hz
1
0
ZX1avg/Controlavg
50 100 300
[ZX1] (μM)
h
5 Hz
10 Hz
1
0
PPR
Control
50 100 300
[ZX1] (μM)
i
IT-PT
25
0
Decay tau (msec)
Control ZX1
j
IT-IT
25
0
Decay tau (msec)
Control ZX1

Figure S5

Figure S5. Synaptic zinc has different potentiating effects on IT-IT synapses and IT-PT synapses at 5 Hz stimulation with no change in PPR. ZX1 does not affect AMPA receptor decay kinetics at IT-PT or IT-IT synapses. Related to Figure 5.

a) Example average IT-IT EPSCs from trains of light pulses at 10 Hz. Magenta: control EPSC. Purple: EPSC after the addition of 50 μM ZX1. Red: EPSC after the addition of 100 μM ZX1. Gold: EPSC after the addition of 300 μM ZX1. Gray: EPSC after the addition of DNQX.

b) Average effect of ZX1 on the average amplitude of IT-IT AMPAR EPSCs, normalized to the corresponding control EPSC amplitudes in response to trains of light pulses at 5 Hz. (50 μM ZX1: p=0.0045, n=11 cells from nine mice; 100 μM ZX1: p=0.3624, n=11 cells from ten mice; 300 μM ZX1: p=0.0114, n=9 cells from nine mice. 2-way repeated measures ANOVA.) Color scheme same as **a.**

c) Average effect of ZX1 on the average amplitude of IT-IT AMPAR EPSCs in response to a 5 Hz stimulus train of blue light normalized to the corresponding control EPSC along the train at each concentration of ZX1 (50 μM ZX1: p=0.0045, n=11 cells from nine mice; 100 μM ZX1: p=0.3624, n=11 cells from nine mice; 300 μM ZX1: p=0.0114, n=9 cells from nine mice. Paired t-test.) Color scheme same as **a.**

d) Average effect of ZX1 on paired pulse ratios at 5 Hz (left) and 10 Hz (right) at IT-IT synapses. (5 Hz: Control: n=21 cells from nineteen mice; 50 μM: p=0.5201, n=11 cells from nine mice; 100 μM: p=0.8052, n=11 cells from ten mice; 300 μM: p=0.0948, n=9 cells from nine mice. 10 Hz: Control n=21 cells from nineteen mice; 50 μM: p=0.8260, n=11 cells from nine mice; 100 μM: p=0.8731, n=11 cells from ten mice; 300 μM: p=0.1270, n=9 cells from nine mice. Paired t-test) Color scheme same as **a.**

e) Example average IT-PT EPSCs from trains of light pulses at 5 Hz. Black: control EPSC. Purple: EPSC after the addition of 50 μM ZX1. Red: EPSC after the addition of 100 μM ZX1. Gold: EPSC after the addition of 300 μM ZX1. Gray: EPSC after the addition of DNQX.

f) Average effect of ZX1 on the amplitude of average IT-PT AMPAR EPSCs normalized to the corresponding control EPSC amplitudes in response to trains of light pulses at 5 Hz. (50 μM ZX1: p=0.0205, n=6 cells from six mice; 100 μM ZX1: p=0.0014, n=12 cells from ten mice; 300 μM ZX1: p=0.0181, n=5 cells from five mice. 2-way repeated measures ANOVA.) Color scheme same as **e.**

g) Average effect of ZX1 on the amplitude of average IT-PT AMPAR EPSCs in response to a stimulus train of blue light normalized to the corresponding control EPSC along the train at each concentration of ZX1 (50 μM ZX1: p=0.0205, n=6 cells from six mice; 100 μM ZX1: p=0.0014, n=12 cells from ten mice; 300 μM ZX1: p=0.0181, n=5 cells from five mice. Paired t-test.) Color scheme same as **e**.

h) Average effect of ZX1 on paired pulse ratios at 5 Hz (left) and 10 Hz (right) at IT-PT synapses. (5 Hz: Control: n=13 cells from eleven mice; 50 µM: p=0.0415, n=6 cells from six mice; 100 µM: p=0.5738, n=12 cells from ten mice; 300 µM: p=0.1284, n=5 cells from five mice. 10 Hz: Control: n=13 cells from eleven mice; 50 µM: p=0.0506, n=6 cells from six mice; 100 µM: p=0.9145, n=12 cells from ten mice; 300 µM: p=0.4219, n=5 cells from five mice. Unpaired t-test.) Color scheme same as in **e**.

i) Average AMPA receptor decay tau at IT-PT synapses. Control (black) compared to post-ZX1 (red). (p=0.2200, n=12 cells from ten mice; paired t-test).

j) Average AMPA receptor decay tau at IT-IT synapses. Control (magenta) compared to post-ZX1 (red). (p=0.6510, n=11 cells from ten mice; paired t-test).

Data from Figure 4g are used in Figure S4f. Asterisks indicate significant p values. Data are represented as mean ± SEM. See Table S1 for detailed statistics.

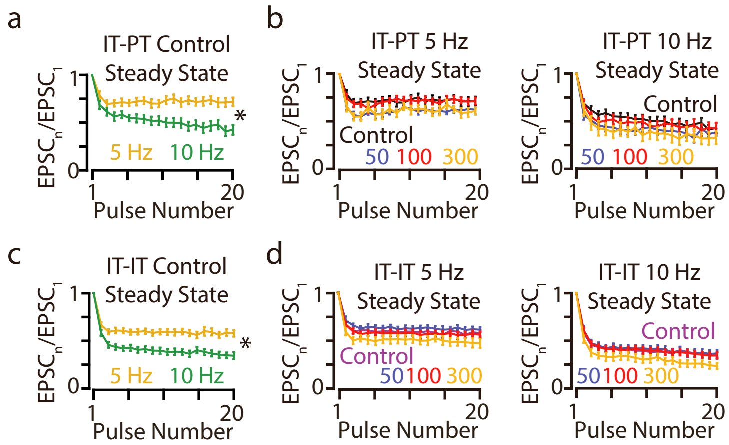

a
IT-PT Control
Steady State
5 Hz 10 Hz
b
IT-PT 5 Hz
Steady State
Control
50 100 300
IT-PT 10 Hz
Steady State
Control
50 100 300
c
IT-IT Control
Steady State
5 Hz 10 Hz
d
IT-IT 5 Hz
Steady State
Control
50 100 300
IT-IT 10 Hz
Steady State
Control
50 100 300
EPSCn/EPSC1
Pulse Number

Figure S6

Figure S6. ZX1 does not alter the frequency-dependent steady-state depression of AMPA EPSC trains at IT-PT or IT-IT synapses. Related to Figures 5 and S5.

a) Average IT-PT control AMPAR EPSC steady state along a stimulus train. Normalized EPSC amplitudes at 5 Hz stimulation (yellow) compared to 10 Hz stimulation (green). (p=2.901e-7, n=13 cells from twelve mice. Two-way repeated measures ANOVA.)

b) Average IT-PT control AMPAR EPSC amplitudes at 5 Hz (left) comparing 50 µM ZX1 (purple), 100 µM ZX1 (red), and 300 µM ZX1 (gold). (5 Hz: Control: n=13 cells from twelve mice. 50 µM ZX1: p=0.0574, n=6 cells from six mice. 100 µM ZX1: p= 0.8273, n=12 cells from twelve mice. 300 µM ZX1: p=0.1202, n=5 cells from five mice. Two-way repeated measures ANOVA.) (Right) Average IT-PT control AMPAR EPSC amplitudes at 10 Hz comparing 50 µM ZX1 (purple), 100 µM ZX1 (red), and 300 µM ZX1 (gold). (10 Hz: Control: n=13 cells from twelve mice. 50 µM ZX1: p=0.1148, n=6 cells from six mice. 100 µM ZX1: p=0.5722, n=12 cells from twelve mice. 300 µM ZX1: p=0.1234, n=5 cells from five mice. Two-way repeated measures ANOVA.)

c) Average IT-IT control AMPAR EPSC steady state along a stimulus train of twenty pulses. Data shown as $EPSC_n/EPSC_1$, normalized EPSC amplitudes compared at 5 Hz stimulation (gold) and 10 Hz stimulation (green). (p=1.9159e-11, n=22 cells from twenty mice. Two-way repeated measures ANOVA.)

d) Average IT-PT control AMPAR EPSC amplitudes at 5 Hz (left) comparing 50 µM ZX1 (purple), 100 µM ZX1 (red), and 300 µM ZX1 (gold). (5 Hz: Control: n=22 cells from twenty mice. 50 µM ZX1: p=0.3742, n=12 cells from eleven mice. 100 µM ZX1: p=0.7365, n=12 cells from twelve mice. 300 µM ZX1: p=0.0892, n=10 cells from nine mice. Two-way repeated measures ANOVA.) Average IT-PT control AMPAR EPSC amplitudes at 10 Hz comparing 50 µM ZX1 (purple), 100 µM ZX1 (red), and 300 µM ZX1 (gold). (10 Hz:Control: n=22 cells from twenty mice. 50 µM ZX1: p=0.7440, n=12 cells from eleven mice. 100 µM ZX1: p=0.9624, n=12 cells from twelve mice. 300 µM ZX1: p=0.3489, n=10 cells from nine mice. Two-way repeated measures ANOVA.)

Asterisks indicate significant p values. Data are represented as mean ± SEM. See Table S1 for detailed statistics.

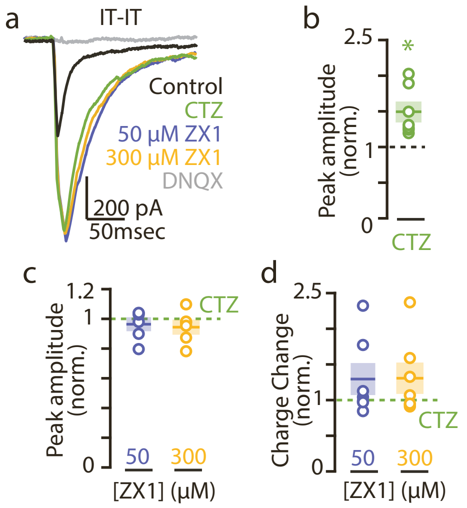
a
IT-IT
Control
CTZ
50 μM ZX1
300 μM ZX1
DNQX
200 pA
50msec
b
2.5
*
Peak amplitude
(norm.)
1
0
CTZ
c
1.2
1
CTZ
Peak amplitude
(norm.)
0
50
300
[ZX1] (μM)
d
2.5
Charge Change
(norm.)
1
CTZ
0
50
300
[ZX1] (μM)

Figure S7

Figure S7. Cyclothiazide blocks the differential enhancement of AMPA receptor function by synaptic zinc at IT-IT synapses. Related to Figure 5.

a) Example traces showing average AMPAR EPSCs in response to a single pulse of blue light. Black: control EPSC. Green: EPSC after the addition of AMPAR agonist cyclothiazide (CTZ). Purple: EPSC after the addition of 50 μM ZX1. Gold: EPSC after the addition of 300 μM ZX1. Gray: EPSC after the addition of DNQX.

b) Average effect of CTZ (green) on the amplitude of average AMPAR EPSCs in response to a single pulse of light ($p=0.00095$, $n=7$ cells from six mice; paired t-test).

c) Average effect of ZX1 on the amplitude of average AMPAR EPSCs in CTZ in response to a single pulse of light. (50 μM ZX1 (purple) vs. CTZ (green): $p=0.4227$, $p=7$ cells from six mice. Unpaired t-test. 300μM ZX1 (gold) vs. CTZ: $p=0.2638$, $n=7$ cells from six mice. Unpaired t-test.)

d) Average effect of ZX1 on the charge of average AMPAR EPSCs in CTZ to trains of light pulses. (50 μM ZX1 (purple) vs. CTZ(green): $p=0.1885$, $n=7$ cells from six mice. Paired t-test. 300μM ZX1 (gold) vs. CTZ: $p=0.1950$, $n=7$ cells from six mice. Paired t-test.)

Asterisks indicate significant p values. Data are represented as mean ± SEM. See Table S1 for detailed statistics.
